# Agents necessitating effects in newtonian time and space: from power and opportunity to effectivity

**DOI:** 10.1007/s11229-018-1769-9

**Published:** 2018-03-27

**Authors:** Jan Broersen

**Affiliations:** Department of Philosophy and Religious Studies, Utrecht University, Utrecht, The Netherlands

**Keywords:** Agency, Time and space, Stit logic, Action theory, Affordances, Powers

## Abstract

We extend *stit* logic by adding a spatial dimension. This enables us to distinguish between powers and opportunities of agents. Powers are agent-specific and do not depend on an agent’s location. Opportunities do depend on locations, and are the same for every agent. The central idea is to define the real possibility to see to the truth of a condition in space and time as the combination of the power and the opportunity to do so. The focus on agent-relative powers and space-relative opportunities firmly roots effectivity of an autonomous choice making agent in a space–time picture. Our space–time view will be classically Newtonian, since we will assume relativistic phenomena do not play a role in agentive effectivity. We show how our semantics naturally distinguishes between different kinds of histories; histories that reflect real (factual) possibilities and histories that reflect counterfactual possibilities (of a particular hypothetical kind). Furthermore, we discuss how the spatial picture sheds light on conceptual problems plaguing the central *stit* property of ‘independence of agency’. At several points in the article we will emphasise and defend the differences with Belnap’s theory of agency in relativistic branching space–times.

## Introduction

Along one dimension of development, *stit* logic has evolved from a theory of agency for general propositional conditions (Kanger 1972; Pörn 1970; Chellas 1995) through a theory of agency for temporal conditions (Horty and Belnap 1995), to a theory of agency for conditions in time and space (Belnap 2005). Like Belnap, we will investigate here the addition of a spatial dimension to temporal *stit* theory. However, our approach is different in many respects, as will be made clear at appropriate points in this article.

There are several motivations for adding a spatial dimension to *stit* logic. One of them is to enable the study of real possibilities of agents in relation to their intrinsic powers and their extrinsic opportunities. In our conceptualisation opportunities introduce a spatial aspect; in Paris agents have the opportunity to climb the Eifel tower,^1^ in Utrecht they do not.^2^ Indirectly this relates to the affordances and potentialities of objects present at these locations (such as the Eifel tower), as we will explain. We hope to show that we can formalise a notion of *situated* agency in terms of real possibilities that are provided by the combination of an agent’s intrinsic *powers* and its extrinsic space related *opportunities*. In a more elaborate picture, agentive powers also play a crucial role in an agent’s *abilities*. However, we leave ability out of the scope here. Ability not only involves agentive power but also *practical knowledge* (Ryle 1946; Anscombe 1963), and we will not consider epistemic or other mental attitudes in this article. Our notion of real possibility-based agency will be without any informational or motivational connotations; it will be a notion of what Marek Sergot calls ‘unwitting agency’ (Sergot 2008).^3^

A second motivation is that in logics of action (e.g., Kowalski and Sergot 1986 or Segerberg 2002) the spatial dimension seems to have been largely neglected. That is a remarkable omission. The temporal dimension plays a role in almost all formalisms of action we are aware of, but apart from Belnap’s proposal we cannot think of one example that also considers a spatial dimension. That is surprising. Why, in logical considerations on agency and action, would one focus on one prominent physical dimension and *not* on the other? Especially in artificial intelligence and robotics a logical theory of actions in time and space is very welcome, it seems safe to claim. We hope this article is a first step towards such a logic of *situated* agents, where ‘situated’ refers to both time and space.

Belnap’s branching space–time theory is based on yet another motivation to involve the spatial dimension in giving formal semantics to agency. His main concern is the issue of *dependency*. Dependency of a situation’s properties on some agent’s choice at some other point in time and space requires that this agent exerts a physical influence of some kind on that situation. This influence has to travel through time *and space*. The subject Belnap pays attention to in particular is then whether or not such an influence on some agent or entity at another location can be *instantaneous*. This relates directly to the interpretation of the central classical *stit* operator. In classical temporal *stit* theory in the Chellas-Belnap-Perloff-Horty tradition the truth condition $\mathcal M, m, h \models [ag \; \mathtt {stit}] \varphi$ for a *stit* formula of the form $[ag \; \mathtt {stit}] \varphi$ says that the agent *ag* in the performance of its current choice along *h*, *sees to it* (necessitates) that *currently* at moment *m* the proposition of the form $\varphi$ is guaranteed to hold.^4^ Belnap observes that $\varphi$ may contain reference to a location that is different from the location of the agent *ag* engaged in the action. A problem Belnap then sees, is that if indeed the agent *ag* featuring in $[ag \; \mathtt {stit}] \varphi$ is at a location other then the location the property $\varphi$ is thought to be made true for, it seems the agentive effect expressed by the truth of $[ag \; \mathtt {stit}] \varphi$ reflects an instantaneous influence by the agent *ag* on some other spatial location of the world it is situated in. That is, if *ag* and $\varphi$ are thought to refer to different locations, we would have that the truth of $[ag \; \mathtt {stit}] \varphi$ reflects an instantaneous effect at a distance of *ag* on $\varphi$. That conflicts with Einstein’s axiom that nothing can travel faster than the speed of light. This motivates Belnap to develop his (truly remarkable) ‘agency in branching space–time theory’ (Belnap 2005). In this work (which we will often refer to as ‘BST-theory’ from now on) Belnap adds agency to his earlier developped relativistic ‘branching space–time’ theory (Belnap 1992). In relativistic space–time there is no absolute reference frame against which we can define simultaneity of events, which implies that effects are local and cannot have instantaneous ramifications at a distance.

We have two reasons not to go along with Belnap’s motivation to add the spatial dimension to *stit* semantics. The first is that we believe that relativistic phenomena of time and space do not seem to be very relevant for agency.^5^ Exactly *because* agency is a very local phenomenon, we should not be bothered about instantaneous effects at large distances being problematic in our relativistic universe. Relativistic effects should only play a role in quite extraordinary circumstances. For reasoning about how two agents in the same room can combine their efforts to lift a table without breaking the precious vase that is on it, relativisitic phenomena seem hardly relevant. What is relevant though in such examples, is what powers these agents have and whether they both actually have the opportunity to lift the table at the right moment (maybe they are too far removed from the table; maybe they lack the strength or agility to move quickly enough, etc.). This is the kind of dependence and interaction relevant for modelling, for instance, artificial agents such as robots. So, at least for the possible use of the formalism we develop here for knowledge representation purposes in Artificial Intelligence, a Newtonian view on space and time should suffice.

Our second reason not to go along with Belnap is that in a *logical* sense, instantaneous effects at a distance should always be possible. This is a difficult point, and we will try to explain it by way of an example. There is no problem with having an instantaneous effect at a distance, if that instantaneous effect is, for instance, of the *logical* form $[\mathtt {X}] p$ saying that (at this distant location) at the *next* moment in time, *p* is guaranteed to be true. There is no risk that this truth constitutes an unwanted physics-defying kind of instantaneous effect at a distance, since the $[\mathtt {X}]$-operator allows for some (unspecified amount of) slack; influence in the form of the necessitation of the proposition *p* can now travel through space *and* time. Now we can say that in such a case *p* is a *non-instantaneous physical* effect at a distance and $[\mathtt {X}] p$ is an *instantaneous logical* effect at a distance. So two spaces play a role, and they should not be confused: logical space and physical space. Logical space is the mathematical dimension we use for logical modelling; we can jump around in it at will and take different viewpoints. Physical space is the space agents perform their actions in. Agents can act and move within this space, but they are bound by its physics. Now why does Belnap not make this distinction? That is not entirely clear. But what is clear, is that his object language has no logical operators for both time and space. So logical operations like jumping to a future moment in time or another location in space are not supported by his language. His language is much closer to the physics of the spaces it studies, which explains why he claims the physical impossibility of instantaneous effects at a distance should be directly reflected by its semantics.

This article sets out then to design a *stit* logic that features both a time and a space dimension and distinguishes between effectivity, power and opportunity. Our setting will be classically Newtonian, and in the next section we will discuss the rammifications this has for our view on branching time.^6^ We present an object language that makes time and space explicit and a formal semantics that enables us to check logical properties of the language. Besides operators for time and space, our object language features operators for power, opportunity and effectivity. Agents have certain powers. But not every situation (location) enables them to use these powers effectively. In our formal logic we show how powers of agents and opportunities of locations are *together* necessary for the generation of *real possibilities* for the agents; possibilities for being *effective* at those locations. The formal semantics of the power and opportunity operators require that we distinguish between different kinds of possibilities. We will distinguish between *factual possibilities* and *counterfactual possibilities*. The first, the factual possibilities, are given semantics by histories in temporal choice structures that reflect courses of events that a situated agent can *effectively* pursue. The second, counterfactual possibilities, reflect on the one hand intrinsic *powers* of agents, powers that might not be effective in certain situations, and on the other hand *opportunities* of *locations*, that can only lead to concrete effects if an agent with the right powers is positioned at that location and executes the right choices. Opportunities are true relative to locations; they are not agentive. Powers are properties of agents. Power and opportunity together determine whether there can be effectivity.

We end this introduction with a very general objection one can have against the kind of enterprise undertaken here. One could claim that logic should not be bothered at all with issues of physical possibility and impossibility. One could take the standpoint that logical theories and physical theories concern different theoretical domains that should be kept entirely separate. In such a view logical truths are independent of physical truths. For instance, logical truths should be consistent with different, mutually inconsistent physical theories (for instance, with both general relativity and quantum mechanics). However, taking this standpoint would dismiss all of temporal logic, action logic, spatial logic, and indeed, *stit* logic. And, in case we endorse a physicalist picture we may argue that it would also dismiss epistemic logic, preference logic, deontic logic, etc. So, we will take a less strict standpoint about the reach of logical validity, one that fits more with the use of logic as a knowledge representation formalism for Artificial Intelligence (AI). In AI, artificial agents use formal logic to represent and reason about their environments. And it seems safe to fix some generally accepted and common physical properties for such environments and take them up as invariants^7^ in a logic.

## Physical and metaphysical considerations on branching

Our starting point is a non-deterministic universe where the truth of properties that refer to the future is not necessarily settled. Alternative temporal trajectories into the future are possible, because the universe inhabits non-deterministic processes allowing for different possible outcomes. One such non-deterministic process is underlying the phenomenon of agency (in a specific way, to be explained below). The alternative trajectories that are possible for the universe from a certain moment, we call ‘branches of time’. One trajectory is an alternative to another trajectory if it obeys different properties, that is, if the non-deterministic processes of our world make other properties of the universe true for that trajectory. So, our view is that branching is *due to* the different possible continuations of the properties of our world: for instance as the result of a radioactive atom decaying or not.^8^ We do not assume that *all* processes in our world are non-deterministic. Or, at least we will assume that some processes are very close to deterministic (without making precise how close exactly). In particular, we will assume the possibility of deterministic processes that keep track of the passing of time: clocks. Clocks cannot be indeterministic processes or else they are no good clocks.

There is a special class of properties that determine which alternative branches are possible as alternative temporal continuations from a given moment. Examples of such properties are dispositions, powers, abilities, opportunities, etc. Branches are different even if they differ only in the sense that they make different such ‘possibility’ properties true.^9^ In this article we look at two kinds of properties influencing possibilities: agentive powers and opportunities.

There is one more aspect to clarify about this basic picture. We may ask: at what point do branches that are different because they obey different properties actually *start* to branch? This is not as simple a question as it may seem. We cannot say that two branches start to branch at the point where their properties start to diverge. This is because in the object language we will use—the language in which we specify the properties—we will be able to refer to future points on branches. Now, if two branches are different because at some point one of them obeys *p* and the other obeys $\lnot p$, with *p* a basic proposition of our language, then also on points that temporally precede that point we will be able to say that they obey different properties, for instance $[\mathtt {X}] p$ and $[\mathtt {X}] \lnot p$. It seems then that we have to say that different branches that are initially undivided start to branch at the point where their *non-temporal* properties start to diverge. A more precise characterisation of this idea is outside the scope of this article.

A suggestion emanating from the above view is that one particular *source* of branching are the choices made by agents. But that is not the right picture for our *stit*-semantics. To get the correct picture it is important first to know that we believe that time branches continuously and massively and not only in relation to the processes that constitute our agency.^10^ This view may seem unlikely to many, but we believe it is the correct picture. It is just that we are easily led to believe that time does not branch, because when looking back in time, we always see exactly only one branch.^11^ Also, by the advancement of science, we become better and better in predicting future properties (think about weather forecasts becoming better). And it may be tempting to belief that this advancement has a limit in the situation where in principle through physical laws and computations we can predict the outcome of any situation.

Our view is *not* that the choices made by agents are yet another source for our massively non-deterministic world; an agentive choice does not *make* time branch. If that would be the correct view, it would suggest that if agents would not choose, there would be less branching. The view we endorse is quite the opposite: if agents would not choose, there would be even more branching. By performing a choice an agent actually stems the flow of branchings of the world by ensuring that all non-deterministic possibilities left by its choice performance obey the effect property that constitutes its action. Describing such an influence on the branching of time as ‘making time branch’, thus puts one on the wrong foot. When misled in this way, one should turn around the picture 180 degrees: agency is about *excluding* non-deterministic possibilities that otherwise *would* be there and is *not* a way to *produce* possibilities that otherwise *would not* be there.

The metaphysical picture gets more complicated if we involve space. We need an idea about what space is and we need to combine it with the branching of time. We will assume that two agents are in the *same* space if and only if their actions *may* interfere ($$=$$ bear the possibility of having joint consequences) at some location in that space at some point in the future.^12^$$^{,}$$^13^ Since we are in a non-deterministic universe, it is not guaranteed that actions of agents in the same space *will* interfere, but the possibility needs to be there in order for them to be in the same space. A consequence of this starting point is that if^14^ time branches for one agent in some space, it also branches for all other agents in that same space. After all, if one agent locally determines some physical properties of some location, at some point in the future, the physical properties of that location may interfere with the physical properties of any other location in that same space. Of course, for rather distant locations the interference may never obtain. But that is not important. What is important is that a choosing agent affects the physical properties of its space *as a whole*. This is not the same as having an effect at a distance. There is an effect at a distance only in the sense that a choice here on Earth has as a result that at Alpha Centauri our common space changes. This effect at Alpha Centauri, we refer to as a *logical* effect. It is not a physical or causal effect. The effect is logical, since the ‘jump’ to the other location at Alpha Centauri is a coordinate jump; we *consider* the same space from another viewpoint and say that also from that viewpoint the physical constellation of our common space has been altered by the choice being made on Earth.

The above view is very different from Belnap’s. Belnap does not distinguish between physical effects and logical effects and just argues that instantaneous effects at a distance are unwanted. In Belnap’s BST-theory if an agent chooses, time branches at the location of the agent, but *not* instantaneously and simultaneously across all other locations of the space the agent inhabites. That is a startling view adding the absence of absolute simultaneity of branching to Einstein’s absence of absolute simultaneity of events. The absence of absolute simultaneity of branching actually follows from the absence of absolute simultaneity of events, as it is the non-determinism of events themselves that makes time branch. In this view it is not the case that for an agent on Alpha Centauri space–time has been altered at exactly the moment where an agent on Earth makes a choice between two alternatives; the point of alternation might be slightly later or earlier, depending on the frame of reference.^15^ We refer to Belnap’s articles for more insights.

We think it is an interesting observation that our distinction between physical and logical effects at a distance, a distinction that is absent in Belnap’s work for reasons already mentioned, seems to allow for a different relativistic interpretation of operators $[ag \; \mathtt {stit}] \varphi$ then Belnap’s. Using our object language it seems we could build a theory that allows for instantaneous *logical* effects at a distance and that at the same time does *not* allow for physical effects at a distance. This theory would then look rather different from Belnap’s, even though, like Belnap’s theory, it would depart from relativistic insights.^16^ We do not pursue that route here though. In the next section we will proceed to give a modal logic account of Newtonian space and time.

## Newtonian branching time and space

Our formal modelling of time and space will be based on some simplifying assumptions. Although we belief, as we said, that time branches massively and *continuously*, we will only consider discrete time where branching occurs in subsequent time steps. For referring to next moments in time, we will introduce the modal quantifier $[\mathtt {X}]$, that reads as ‘next’. A second reason to consider the next operator instead of a conceptually less demanding operator like the well-known temporal ‘henceforth’ operator, is that we can use it to capture an important property of Newtonian time and space: the reality of *global clocks*. We can use the next operator to capture this property, since the next can be used for ‘timing’ purposes; we are going to assume that every step interpreting some instance of the next operator has a sufficiently small (for scenario modelling purposes) and *equal* duration. To refer to the other branches that emanate from the same moment (and location) we use the modal quantifier $[\mathtt {H}]$ that reads as ‘it is historically necessary/it is settled’. Besides these familiar operators we introduce a new modal universal quantifier $[\mathtt {L}]$ that ranges over all positions/locations in the current space. The spatial operator enables us to express that something is true independent of the location in the current space ($[\mathtt {L}]$) and to express that there is a location in the present space for which something is true ($\langle \mathtt {L} \rangle$). This is the simplest kind of location operator one can think of. Yet it is complex enough to provide us with some serious modelling problems, both conceptually and technically. For the purpose of this article, the issues of topology, number of dimensions and structure of the locations are not relevant; we will treat locations as unanalysed points whose topology has no bearing on the issues considered here. In particular, in order to represent the distinction between power and opportunity in Sect. 4, we will not need to dig into the structure of space itself: a point-like representation of locations will do the job.

### Definition 3.1

(*Language*) Given a countable set of propositions *P* and $p \in P$, and given a finite set *Ags* of agent names, and $ag \in Ags$, the formal language $\mathcal L_{\textsf {NBTS}}$ is:

$$\begin{aligned} \begin{array}{lcl} \varphi &{} := &{} p \mid \lnot \varphi \mid \varphi \wedge \varphi \mid [\mathtt {L}] \varphi \mid [\mathtt {X}] \varphi \mid [\mathtt {H}] \varphi \\ \end{array} \end{aligned}$$

For all operators the reading is relative to a position in time and space. We call such a position in time and space a ‘situation’. And we say that truth is ‘situated’. The readings are as follows.

- $[\mathtt {L}] \varphi$ reads as “for all locations (in the evaluation space), at the moment of evaluation, along the history of evaluation, it holds that $\varphi$”. We will assume that the locations visible from different situations are always the same set; so there is only one space.
- $[\mathtt {X}] \varphi$ reads “at the next moment, along the history of evaluation, at the location of evaluation, it holds that $\varphi$”. We can think of the next moment in time as being specified by an implicit clock related to a specific deterministic process. The granularity of the succession of times is arbitrary: we can make it as fine-grained as needed for a reasoning scenario.
- $[\mathtt {H}] \varphi$ reads “whatever happens, it holds that/it is historically necessary that/it is settled that at the moment and location of evaluation $\varphi$”.

We introduce the spatial dimension in the same way as histories and strategies are introduced in *stit* logic: as elements of the units of evaluation. This makes truth location dependent/situated. Concrete examples of truths that are location dependent are: “it is safe”, “it is raining”, “one can see the Eiffel tower”, etc. Examples of truths that are not location dependent are: “today is Mieke’s birthday”, “Mieke can play guitar”, “Mieke’s house is burning”, etc. Sentences like “the house is burning” might confuse us though, because we can either interpret “the” relative to a location or not. Truth evaluation relative to locations is very natural; we can always view an agent as being situated in some space evaluating the propositions he is reasoning about relative to locations of the space around him/her. Like temporal extensions of Chellas *stit* logic make truth relative to histories, spatial extensions make truth relative to locations.

**Fig. 1:**
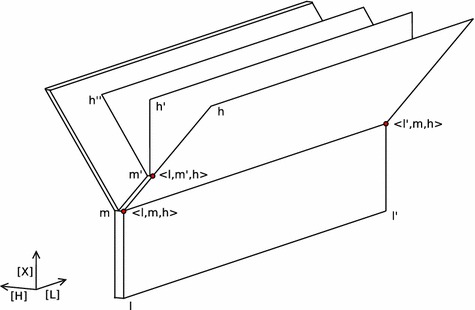
Visualisation of Newtonian branching time and space

For helping to create a mental picture in which to understand the coming definitions, in Fig. 1, we provide a visualisation of Newtonian time and space. The figure shows, for instance, how at moment $m'$ the future branches out into three histories *h*, $h'$ and $h''$, and that this branching is uniform over space, stretching from *l* to $l'$. Points in this three dimensional space will be called ‘situations’ and are denoted $\langle l,m,h \rangle$. The picture highlights three such situations.

### Definition 3.2

(*Frames*) An NBTS-frame is a tuple $\langle L, M, H, \mathcal {L}, \mathcal {X}, \mathcal {H} \rangle$ such that^17^:

1.: *L* is a non-empty set of locations. Elements of *L* are denoted *l*, $l'$, etc.
2.: *M* is a non-empty set of moments. Elements of *M* are denoted *m*, $m'$, etc.
3.: *H* is a non-empty set of possible system histories. Each individual history $h \in H$ is of the form $< \!\! \ldots m_{-2}, m_{-1}, m_{0}, m_{1}, m_{2}, \ldots \!\!>$ with $m_x \in M$. Each history *h* is isomorphic to the integers $\mathbb {Z}$ under an isomorphism $f_h$ such that $f_h(m_{i + 1}) = f_h(m_{i}) + 1$.
  We define the h-relative ‘next moment’ functions $next_h: M \mapsto M$ such that $next_h(m) = m'$ if and only if $m,m' \in h$ and $f_h(m) + 1 = f_h(m')$.
  Histories can come together only in the ‘past’ direction, and if they do, they stay together going further into the past. So, if for $h, h' \in H$ it holds that $next_h(m) = next_{h'}(m')$ then $m = m'$.
  We define **situations** as tuples $\langle l,m,h \rangle$, with $l \in L$, $m \in M$, $h \in H$. A situation will be called **coherent** if $m \in h$ and **incoherent** if $m \not \in h$. In the sequel, when we refer to a situation, we will always mean a ‘coherent situation’; **incoherent situations will not be considered**.
4.: $\mathcal {L}$ is a spatial reference relation over situations. We have the following constraint ensuring reference to other locations using the location operator does not affect the moment or history component of a situation (that is, spatial reference is a logical operation, not a physical one involving ‘movement’):

- **independence of space:**$\langle l,m,h \rangle \mathcal {L} \langle l',m',h' \rangle$ if and only if $m= m'$ and $h= h'$

5.: $\mathcal {X}$ is a next-time relation over situations. We have that:

- **independence, seriality and functionality of time steps:**$\langle l,m,h \rangle \mathcal {X} \langle l',m',h' \rangle$ if and only if $l= l'$ and $h= h'$ and if $next_h(m) = m'$

6.: $\mathcal {H}$ is a cross-history relation over situations. We have the following constraint ensuring reference to other situations using the cross-history operator does not affect the moment or location component of a situation:

- **independence of branching:**$\langle l,m,h \rangle \mathcal {H} \langle l',m',h' \rangle$ if and only if $m= m'$ and $l= l'$

**Ad 3**: Note that moments can be shared by different histories, and if they do, there must be a particular moment from where the histories stay together into the past direction and branch out into strictly separate directions into the future (if there is not, either the histories are not different or do not share a moment at all). This is their branching point. There can also be moments where 3 or more (up until countably many) histories have their branching point, such as in moment $m'$ in Fig. 1.

**Ad 3’**: Since we assume histories are isomorphic with the integers and since we assume time-space is not relativistic, we have a rudimentary notion of ‘time’ in the semantics; from any given moment, we can count steps into the future and into the past and assume that each step has the same duration. For practical modelling purposes it seems we can always choose the duration of time steps sufficiently small to capture the scenario at hand. This construction thus shows clearly that moments should not be *identified* with ‘times’; if at a certain moment and time for the next instance of time there are two diverging possible temporal continuations (histories), then there are two possible next *moments* but there is only one next *time*.^18^ We do not make times explicit in the object language which explains why times play no significant role in our logical framework.

**Ad 3”**: We define a ‘bundle’ of histories as a set $B \subseteq H$ such that for any two $h, h' \in B$ it holds that $h \cap h' \not = \emptyset$. One may wonder if we should not impose more constraints on our branching structures. For instance, we could impose that *H* itself is a bundle. This would mean that any two histories in *H* at some moment in backwards temporal direction meet. However, conditions like these will not be important for us, since the modal language we will use to talk about the structures is not strong enough to express them.

### Definition 3.3

(*Models*) A frame $\langle L, M, H, \mathcal {L}, \mathcal {X}, \mathcal {H} \rangle$ is extended to a model $\langle L, M, H, \mathcal {L}, \mathcal {X}, \mathcal {H}, V \rangle$ by adding a valuation *V* of atomic propositions:

- *V* is a valuation function $V: P \mapsto 2^{L \times M \times H}$ assigning to each atomic proposition the set of situations relative to which they are true.

Note that that we have assumed that all situations are coherent (see Definition 3.2, after item 3), so this also holds for the situations $\langle l, m, h \rangle$ in the above definition and in the definitions yet to come. We will now define the truth conditions for the operators relative to coherent situations in NBTS-frames.

### Definition 3.4

(*Truth*) Relative to a model $\langle L, M, H, \mathcal {L}, \mathcal {X}, \mathcal {H}, V \rangle$, truth $\mathcal \langle l, m, h \rangle \models \varphi$ of a formula $\varphi$ in a space–time situation $\langle l, m, h \rangle$ with $m \in h$ is defined as^19^

$$\begin{aligned} \begin{array}{lll} \mathcal \langle l, m, h \rangle \models p &{} \Leftrightarrow &{} \langle l,m,h \rangle \in V(p) \\ \mathcal \langle l, m, h \rangle \models \lnot \varphi &{} \Leftrightarrow &{} \text{ not } \mathcal \langle l, m, h \rangle \models \varphi \\ \mathcal \langle l, m, h \rangle \models \varphi \wedge \psi &{} \Leftrightarrow &{} \mathcal \langle l, m, h \rangle \models \varphi \text{ and } \mathcal \langle l, m, h \rangle \models \psi \\ \mathcal \langle l, m, h \rangle \models [\mathtt {L}] \varphi &{} \Leftrightarrow &{} \langle l, m, h \rangle \mathcal {L} \langle l', m', h' \rangle \text{ implies } \mathcal \langle l', m', h' \rangle \models \varphi \\ \mathcal \langle l, m, h \rangle \models [\mathtt {X}] \varphi &{} \Leftrightarrow &{} \langle l, m, h \rangle \mathcal {X} \langle l', m', h' \rangle \text{ implies } \mathcal \langle l', m', h' \rangle \models \varphi \\ \mathcal \langle l, m, h \rangle \models [\mathtt {H}] \varphi &{} \Leftrightarrow &{} \langle l, m, h \rangle \mathcal {H} \langle l', m', h' \rangle \text{ implies } \mathcal \langle l', m', h' \rangle \models \varphi \\ \end{array} \end{aligned}$$

Satisfiability, validity on a frame and general validity are defined as usual.

### Definition 3.5

(*Hilbert system*) The following axiom schemas, in combination with a standard axiomatization for propositional logic, and the standard rules *(*like necessitation*)* for the normal modal operators ‘$[\;]$’ define the Hilbert system $\mathsf {NBTS}_{Hilb}$

$$\begin{aligned} \begin{array}{ll} \text{(normal } \text{ modal } \text{ logic) } &{} \text{ S5 } \text{ for } [\mathtt {H}] \\ \text{(normal } \text{ modal } \text{ logic) } &{} \text{ S5 } \text{ for } [\mathtt {L}] \\ \text{(normal } \text{ modal } \text{ logic) } &{} \text{ Alt.D } \text{ for } [\mathtt {X}] \qquad \qquad (\text{ obeying } \text{ e.g. } \lnot [\mathtt {X}] \lnot \varphi \leftrightarrow [\mathtt {X}] \varphi ) \\ \text{(global } \text{ clocks) } &{} [\mathtt {X}][\mathtt {L}] \varphi \leftrightarrow [\mathtt {L}][\mathtt {X}] \varphi \\ \text{(global } \text{ branching) } &{} [\mathtt {H}][\mathtt {L}] \varphi \leftrightarrow [\mathtt {L}][\mathtt {H}] \varphi \\ \text{(forward } \text{ branching) } &{} [\mathtt {H}] [\mathtt {X}] \varphi \rightarrow [\mathtt {X}] [\mathtt {H}] \varphi \\ \end{array} \end{aligned}$$

Relative to a language $\mathcal L$, a class of frames $\mathcal F$ and a truth definition $\models$, we define the logic $Logic(\mathcal F)$ of a class of frames $\mathcal F$ as the subset $Logic(\mathcal F) \subseteq \mathcal L$ of the formulas of the language that are generally valid for the class of frames according to the truth definition.

### Proposition 3.1

(Soundness) $$Logic(\textsf {NBTS}\text{-frames }) \supseteq \mathsf {NBTS}_{Hilb}$$

### Conjecture 3.1

(Completeness) $$Logic(\textsf {NBTS}\text{-frames }) \subseteq \mathsf {NBTS}_{Hilb}$$

### Sketch of a proof

All axioms are in the Sahlqvist class. This leaves us to prove that the first-order correspondents of the axioms correspond to the constraints we have put on our frames. This is a doable exercise. Construe a one-to-one correspondence between situations $\langle l, m, h \rangle$ and possible worlds *w* such that $w = w'$ if and only if $l =l'$ and $m=m'$ and $h=h'$. This transforms the structures into standard one-dimensional structures with multiple modalities. The constraints on the original three-dimensional frames can now be rewritten into standard first-order constraints on the newly constructed one-dimensional structures. These first-order conditions then correspond to the Sahlqvist axioms. Note that the conditions to be checked are all standard ones: equivalence, functionality and commutativity.

Within the context of the other axioms ensuring e.g. functionality for the $[\mathtt {X}]$ operator, the forward branching axiom corresponds to the condition in Definition 3.2 clause 3 saying that there can be no divergence of histories in a backwards time direction; if for two histories $h = < \!\! \ldots m_{1}, m_{2}, \ldots \!\!>$ and $h' = < \!\! \ldots m_{3}, m_{4}, \ldots \!\!>$ it holds that $m_{2} = m_{4}$ and *not*$m_{1} = m_{3}$, then $\langle \mathtt {X} \rangle \langle \mathtt {H} \rangle p \rightarrow \langle \mathtt {H} \rangle \langle \mathtt {X} \rangle p$, with *p* an atomic proposition, is easy to falsify in a *p*-based model in a coherent situation based on the moment $m_1$.

The axiomatisation can do without confluency axioms $\langle \mathtt {X} \rangle [\mathtt {L}] \varphi \rightarrow [\mathtt {L}] \langle \mathtt {X} \rangle \varphi$ and $\langle \mathtt {H} \rangle [\mathtt {L}] \varphi \rightarrow [\mathtt {L}] \langle \mathtt {H} \rangle \varphi$, as these are derivable (we only need symmetry for *one* of the operators involved in these formulas to derive confluency from commutativity, and symmetry follows in S5 for $[\mathtt {L}]$). $\square$

The fragment of our logic determined by the operator $[\mathtt {X}]$ is Segerberg’s ‘tomorrow’ logic (Segerberg 1967). A more recent name for the logic is *Alt*.*D* (*Alt* for partial functionality and *D* for seriality).

The reason we present completeness as a conjecture is that it is not 100% guaranteed the proof strategy sketched above can be successfully employed. It is possible that the class of structures characterised by the Sahlqvist axioms is still a superset of the structures of Definition 3.2. In particular it might be the case that Definition 3.2 imposes stronger interactions between the dimensions than is reflected by the commutativity axioms. However, it is important to stress that the three base dimensions of time ($[\mathtt {X}]$), space ($[\mathtt {L}]$) and branching ($[\mathtt {H}]$) do *not* form the three dimensional product logic^20^$S5 \times S5 \times Alt.D$. Confronted with the semantic clauses in Definition 3.2—4, 5 and 6 one could easily be led to belief this, though. Indeed the space dimension forms a two dimensional product with both the time and the branching dimension, but the interaction between the time and the branching dimension is weaker (which does *not* mean that if this latter interaction actually was also a product, the three dimensions *would* form a three-dimensional product). This is because our logic only concerns *coherent* situations $\langle l,m,h \rangle$ for which $m \in h$. This dictates that a moment *m* and a history *h* cannot be chosen independently, as would be required for a full three-dimensional product frame. Actually, what we have is a three dimensional ‘relativized’ product (Kurucz 2007). Then, more circumstantial evidence for the completeness is that most relativized products inherit the meta-logical properties of the logic formed by the *fusion* of their dimensions (Kurucz and Zakharyaschev 2003). We will however refrain from trying to give a detailed proof. The envisioned audience of this paper is not mathematical logicians, but philosophers of agency, time, and powers.

It can be a surprisingly impactful step to go from two to three dimensions.^21^ Two S5 dimensions combined in a ‘coordinate system’-type-of-way are fully axiomatised by a commutativity axiom, as exemplified by our pairs of dimensions $[\mathtt {L}] \times [\mathtt {X}]$ and $[\mathtt {L}] \times [\mathtt {H}]$. But *three* S5 dimensions combined in a ‘coordinate system’-type-of-way are *not* finitely axiomatisable^22^ and their logic is *not* decidable. One should not underestimate the ontological relevance of such unexpected ‘jumps’ in logical characterisability. If a logical language reflects our capacity to talk about the kind of structures we study, then absence of axiomatisability and undecidability (of the satisfiability problem) for the logic defined relative to this language imply that we will not always be able to (formally, computationally) check if what we express in our language actually has a model. And, if indeed there would be no model for what we say in terms of the language, our talk is, at least from one important point of view, meaningless.^23^

## Power and opportunity

The previous section formalises how branching interacts with space, and we adopted a classical Newtonian view on this. In this section we add to the picture how branching relates to powers of agents and to opportunities at specific locations in space. Individual powers and opportunities are interpreted by sets of branches: the histories the possible future continuations of the world is restricted to, according to that particular power or opportunity. But first we need to describe in more detail what our power and opportunity concepts are.

We view powers as properties of agents. We are aware that the literature has adopted a more general use of the term, where powers are properties of physical objects in general; being ascribed to agents, trees and stones alike (see e.g., Mayr 2011). We believe our use of the term ‘power’ as an agentive property is closer to common natural language use; we feel it is somewhat odd to talk of the powers of stones, since inanimate objects like stones do not *act*. An alternative would have been to use the term ‘capacities’, which has a strong agentive flavour to it. But, the connotation of capacities as being a quantitive measure makes this in our opinion a less suitable term; our agentive powers are *qualitative*.

Also our use of the term ‘opportunities’ requires further explanation. In our conceptualisation opportunities are relative to places and moments. They are independent of agents, and thus, of histories. Opportunities, in this view, encode what futures are possible as seen from a particular point in space. For instance, standing on top of the Utrecht Dom tower, with little effort I can drop a stone on the Utrecht academy building. But, standing in that place, I cannot with little effort drop a stone on the Utrecht railway station. The opportunities of locations (and moments) are determined by the *affordances* or *potentialities* of objects present at these locations and their direct surroundings (at a particular moment). In the stone-throwing example: the opportunity to throw a stone at the academy building hinges on the position at the Dom tower one has and the availability of a suitable stone there (not too heavy, not too light).

Affordances originate in behavioural psychology but are now also studied in robotics (Chemero and Turvey 2007) and virtual environments.^24^ There are different competing theories about the nature of affordances that differ on whether they are objective properties or subjective properties (Gibson 1979 vs. Norman 1999) or on whether they have an identity independent of agents or not (Turvey 1992 vs. Chemero 2003). The affordances as we prefer to interpret them are objective (in line with Gibson) and have a physical identity independent of agents (in line with Turvey). Potentialities are talked about by Aristotle who distinguishes them form actualities. Barbara Vetter describes potentialities as follows (Vetter 2015): “Potentialities are possibilities rooted in objects; they are like possibilities, but they are properties of individual objects.”.

However, our theory is not about affordances and potentialities of objects directly, but about the opportunities of specific locations they give rise to indirectly through their situatedness in time and space. Our view here is that the objects present at locations and their surroundings, through their affordances and potentialities, determine the opportunities of that location. And the opportunities of different locations, they all hang together. But, there is no systematic logical way in which they do. The way they hang together is determined by the physical constellation of our world at the moment of consideration. The physical constellation, in turn, is determined by the affordances of objects present at neighbouring locations.

We can question this view in several ways. A first possible objection is that it seems we might have overlooked certain physical conditions of locations that influence their opportunities. To go back once more to the rock-throwing example: the opportunity to throw a stone at the academy building also hinges on physical conditions like the wind being not too strong and/or coming from the right direction and gravity being gravity as we normally experience it on earth. However, as we see it, also these conditions result from affordances of objects in the surroundings. The gravity we normally experience can be seen as an affordance of our planet. Properties of the wind are properties of the ‘air objects’ in our atmosphere.

A second objection is that according to physics it is incorrect to allocate properties to points in *space*. Since Einstein, we know that space is not an absolute reference frame against which we can evaluate the truth of contingent physical properties. Yet, that is what we propose to do here. As we already explained, our assumption is that for the study of the phenomenon of agency this is justified. And under this assumption—the absoluteness of space—we can see physical opportunities indeed as properties of reference points in space.

A third objection is that there are kinds of opportunities that are not at all linked to space or objects in space. These are typically (and maybe exclusively) opportunities related to situations in social reality (Searle 1995) instead of physical reality. Examples are opportunities related to social position, money and trades, rights and permissions, etc. For instance, one only gets the opportunity to marry the person one has loved for a long time if that person divorces her/himself from her/his current partner. One only gets the opportunity to own a house in the center of Amsterdam as soon as one can afford it (if ever). And one only gets the opportunity to go to university if one successfully finished high school. Our ideas may be transferable to *situatedness in social reality*, but we leave this investigation for future research.

Finally we might object against the whole enterprise of making a distinction between powers and opportunities. Maybe it is not always very clear what exactly the distinction between powers and opportunities is; does an agent who steps into a very fast car obtain the *power* to drive very fast or the *opportunity* to drive very fast? Our answer in that case is that the agent obtains the opportunity, and not the power, because our notion of power is independent of locality. But there are other cases where the distinction is not so clear. For instance, things are already less clear if we think about the power/opportunity to see clearly only if one wears glasses. We believe that ‘opportunity’ is still the right modality here, but one can see where this is going; from the glasses we can turn our attention to our eyes, our optic nerves, etc. Here we will not address this issue any further and will assume that we *can* make a sensible distinction between power and opportunity.

Let us now try to make these ideas formal. We do this by adding power and opportunity operators to the language of Newtonian space and time of the previous section. The following definition gives the extended syntax.

### Definition 4.1

Given a countable set of propositions *P* and $p \in P$, and given a finite set *Ags* of agent names, and $ag \in Ags$, the formal language $\mathcal L_{\textsf {NBTS.PO}}$ is:

$$\begin{aligned} \begin{array}{lcl} \varphi &{} := &{} p \mid \lnot \varphi \mid \varphi \wedge \varphi \mid [\mathtt {L}] \varphi \mid [\mathtt {X}] \varphi \mid [\mathtt {H}] \varphi \mid \langle [ag \; \mathtt {pwr}] \rangle \varphi \mid \langle [\mathtt {oppt}] \rangle \varphi \\ \end{array} \end{aligned}$$

The power and opportunity operators have the following readings.

- $\langle [ag \; \mathtt {pwr}] \rangle \varphi$ reads “agent *ag* at the moment of evaluation has the power to see to it that $\varphi$”.
- $\langle [\mathtt {oppt}] \rangle \varphi$ reads “at the moment and location of evaluation there is an opportunity to see to it that $\varphi$”.

It is important to read $\langle [ag \; \mathtt {pwr}] \rangle \varphi$ correctly. It will therefore be good to consider some readings that are wrong. One incorrect reading is “*ag* is at the location of evaluation, where it has the power for $\varphi$”. So, truth of $\langle [ag \; \mathtt {pwr}] \rangle \varphi$ does *not* say the agent *ag* is actually present at the location of evaluation. Another incorrect reading would be “wherever *ag* is, it has the power for $\varphi$ at the location of evaluation”. This is incorrect, because for most properties $\varphi$, relative to the location of evaluation, it *does* matter where the agent is: for non-spatial properties $\varphi$ it actually needs to be present at the location of evaluation, and for other properties, it may need to be present at some specific other location in space. The correct reading is thus as stated above. If we want to make it more precise, but also cumbersome, we could correctly change it to “from its unknown location somewhere in space (that is possibly not the location of evaluation) *ag* has the power for $\varphi$ at the location of evaluation”. By this reading we avoid talking about locations of agents in our object language. If we would have wanted to talk about locations of agents in the object language, we would also have to assign locations to agents in the structures. From a logic perspective it seems preferable not to do that.

One feature that distinguishes powers from opportunities is that they are uniform over space. This property adds further flavour to the above explained reading of the power operator. We explained how operators should be interpreted as not saying anything about the location of agents. Now we add as an extra condition that wherever an agent is, it has the same powers. So, metaphorically speaking, we could say that the power operator ‘hides’ an agent’s location.

In contrast to powers, opportunities are strictly ‘local’ in the sense that they encode how local conditions affect what is possible globally. An opportunity here in Utrecht restricts what is possible in Amsterdam in the sense that the possible histories of both cities’ common space are restricted to the ones compatible with the opportunities here in Utrecht. Yet, still these are the opportunities of Utrecht, and not of Amsterdam; if Amsterdam’s opportunities were changed, and Utrecht’s opportunities not, then we would induce another subdivision of the global histories admitted by Utrecht’s opportunities.

We extend the NBTS-frames $\langle L, M, H, \mathcal {L}, \mathcal {X}, \mathcal {H} \rangle$ of Definition 3.2 to NBTS.PO-frames $\langle L, M, H, \mathcal {L}, \mathcal {X}, \mathcal {H}, \mathcal {P}, \mathcal {O} \rangle$ that include structure for powers and opportunities.

### Definition 4.2

An NBTS.PO-frame is a tuple $\langle L, M, H, \mathcal {L}, \mathcal {X}, \mathcal {H}, \mathcal {P}, \mathcal {O} \rangle$ such that^25^:

1.: $\langle L, M, H, \mathcal {L}, \mathcal {X}, \mathcal {H} \rangle$ is an NBTS-frame.
2.: $\mathcal {P}: M \times Ags \mapsto 2^{2^{H}}$ is a power function yielding for any moment and agent a set of powers. We apply the following constraints.
  a.: **moment specificity of powers:** if $P \in \mathcal {P}(m,ag)$ and $h \in P$ then $m \in h$
  b.: **power for historical necessities:** $\emptyset \not = \mathcal {P}(m,ag)$
  c.: **no power for historical impossibilities:** $\emptyset \not \in \mathcal {P}(m,ag)$
  d.: **powers induce branching:** if $P \in \mathcal {P}(m,ag)$ and $h \in P$ and $h' \in H$ and $m \in h'$ and $h' \not \in P$, then $next_h(m) \not = next_{h'}(m)$
3.: $\mathcal {O}: L \times M \mapsto 2^{2^{H}}$ is an opportunity function yielding for any location and moment a set of opportunities. We apply the following constraints.
  a.: **moment specificity of opportunities:** if $O \in \mathcal {O}(l,m)$ and $h \in O$ then $m \in h$
  b.: **opportunity for historical necessities:** $\emptyset \not = \mathcal {O}(l,m)$
  c.: **no opportunity for historical impossibilities:** $\emptyset \not \in \mathcal {O}(l,m)$
  d.: **opportunities induce branching:** if $O \in \mathcal {O}(l,m)$ and $h \in O$ and $h' \in H$ and $m \in h'$ and $h' \not \in O$, then $next_h(m) \not = next_{h'}(m)$

**Ad 2**: Individual powers are sets of histories. A set of histories modelling a power reflects one possible impact an agent can have on the properties of the world; the agent can ensure the actual history is among the members of the set.^26^ Powers are not relative to locations or histories, but only to moments and agents. Note that this definition hinges crucially on the fact that histories are uniform across space, which would not have been the case if we would have adopted relativistic space–time. Powers do depend on moments, to reflect that the powers of an agent may change over time.

NBTS structures do *not* provide a location of the agent somewhere is space. We already explained that the reading of object level formulas is such that their truth value does not depend in any relevant way on the location of the agent in space. This explains why the structures do not provide that information.

**Ad 3**: Individual opportunities are sets of histories. A set of histories representing an opportunity reflects one possible impact an agent with suitable powers could have on the course of events of the world as seen from a certain location. Opportunities are not relative to agents or histories, but only to locations and moments.

Conditions 2.d. and 3.d. are *essential* to bring the branching of histories in connection with the exertion of powers and the exploitation of opportunities. Without these conditions, powers and opportunities would be mere sets of subsets of histories that have no connection with their branching structure. The conditions ensure that powers and opportunities actually differentiate between different possible temporal continuations of a moment. These properties are similar to the well known ‘no choice between undivided histories’ condition from *stit*-theory (see the discussion after Definition 5.2 in the next section).

Note that opportunities and powers are *independent* influencers of a situation’s possible futures, that is, if in a certain situation something is an opportunity there is no reason for it to also be a power, and if something is a power there is no reason for it to also be an opportunity. But, as we will define in the next section, both qualities are needed to enable real possibilities. This means that powers and opportunities themselves are not given semantics through histories that necessarily reflect what is actually (or really) possible in the world. Only the histories interpreting the agentive effectivity function defined in the next section represent real possibilities. This raises the question what the exact ontological status is of the histories implementing powers and opportunities. Our answer is that they are counterfactual possibilities of a specific kind. So, our theory distinguishes between real (or factual) possibilities^27^ and counterfactual possibilities.

To understand the definition of opportunities as subsets of histories at specific locations better, let us come back to the Utrecht–Amsterdam example once more. Even though the subsets of histories determined by Utrecht’s opportunities are also ‘present’ in Amsterdam (as explained, in our Newtonian setting histories branch out uniformly over space, see Fig. 1) they cannot be ‘exploited’ from the Amsterdam perspective; they have to be exploited by an agent from the Utrecht perspective: Utrecht’s opportunities are not the same as Amsterdam’s opportunities.

We will now define a logic of power and opportunity in the space and time setting of Sect. 3. The logic will be defined semantically. A Hilbert system based on sound principles is also provided, but we do not know if it characterises the logic completely. We will discuss valid schemas and non-valid schemas, to sharpen our intuitions about the system and validate its modelling adequacy.

### Definition 4.3

Relative to a model $\langle L, M, H, \mathcal {L}, \mathcal {X}, \mathcal {H}, \mathcal {P}, \mathcal {O}, V \rangle$, truth $\mathcal \langle l, m, h \rangle \models \varphi$ of a formula $\varphi$ in a space–time situation $\langle l, m, h \rangle$ with $m \in h$ is defined as:

$$\begin{aligned} \begin{array}{lll} \langle l, m, h \rangle \models \langle [ag \; \mathtt {pwr}] \rangle \varphi &{} \Leftrightarrow &{} \exists P \in \mathcal {P}(m,ag) \text{ such } \text{ that } \forall h' \in P, \langle l, m, h' \rangle \models \varphi \\ \langle l, m, h \rangle \models \langle [\mathtt {oppt}] \rangle \varphi &{} \Leftrightarrow &{} \exists O \in \mathcal {O}(l,m) \text{ such } \text{ that } \forall h' \in O, \langle l, m, h' \rangle \models \varphi \\ \end{array} \end{aligned}$$

Satisfiability, validity on a frame and general validity are defined as usual.

### Definition 4.4

The following axiom schemas, in combination with a standard axiomatization for propositional logic, and the standard rules *(*like necessitation*)* for the normal modal operators ‘$[\;]$’, and the non-normal operators ‘$\langle [\; ] \rangle$’ define the Hilbert system $\mathsf {NBTS.PO}_{Hilb}$

$$\begin{aligned} \begin{array}{ll} \text{ Power } &{} \\ \text{(moment } \text{ determinacy } \text{ of } \text{ power) } &{} \langle [ag \; \mathtt {pwr}] \rangle \varphi \rightarrow [\mathtt {H}] \langle [ag \; \mathtt {pwr}] \rangle \varphi \\ \text{(power } \text{ for } \text{ historical } \text{ necessities) } &{} [\mathtt {H}] \varphi \rightarrow \langle [ag \; \mathtt {pwr}] \rangle \varphi \\ \text{(no } \text{ power } \text{ for } \text{ historical } \text{ impossibilities) } &{} [\mathtt {H}] \varphi \rightarrow \lnot \langle [ag \; \mathtt {pwr}] \rangle \lnot \varphi \\ \text{(location } \text{ independence } \text{ of } \text{ power) } &{} \langle \mathtt {L} \rangle \langle [ag \; \mathtt {pwr}] \rangle \varphi \leftrightarrow \langle [ag \; \mathtt {pwr}] \rangle \langle \mathtt {L} \rangle \varphi \\ \text{(monotonicity } \text{ of } \text{ power) } &{} \langle [ag \; \mathtt {pwr}] \rangle (\varphi \wedge \psi ) \rightarrow \langle [ag \; \mathtt {pwr}] \rangle \varphi \\ \text{(reduction } \text{ of } \text{ second-order } \text{ power) } &{} \langle [ag \; \mathtt {pwr}] \rangle \langle [ag \; \mathtt {pwr}] \rangle \varphi \rightarrow \langle [ag \; \mathtt {pwr}] \rangle \varphi \\ \text{(reduction } \text{ of } \text{ double } \text{ power } \text{ negation) } &{} \lnot \langle [ag \; \mathtt {pwr}] \rangle \lnot \langle [ag \; \mathtt {pwr}] \rangle \varphi \rightarrow \langle [ag \; \mathtt {pwr}] \rangle \varphi \\ \text{(powers } \text{ induce } \text{ branching) } &{} \langle [ag \; \mathtt {pwr}] \rangle [\mathtt {X}] \varphi \rightarrow \langle \mathtt {H} \rangle [\mathtt {X}] [\mathtt {H}] \varphi \\ &{} \\ \text{ Opportunity } &{} \\ \text{(moment } \text{ determinacy } \text{ of } \text{ opportunity) } &{} \langle [\mathtt {oppt}] \rangle \varphi \rightarrow [\mathtt {H}] \langle [\mathtt {oppt}] \rangle \varphi \\ \text{(opportunity } \text{ for } \text{ historical } \text{ necessities) } &{} [\mathtt {H}] \varphi \rightarrow \langle [\mathtt {oppt}] \rangle \varphi \\ \text{(no } \text{ opportunity } \text{ for } \text{ historical } \text{ impossibilities) } &{} [\mathtt {H}] \varphi \rightarrow \lnot \langle [\mathtt {oppt}] \rangle \lnot \varphi \\ \text{(monotonicity } \text{ of } \text{ opportunity) } &{} \langle [\mathtt {oppt}] \rangle (\varphi \wedge \psi ) \rightarrow \langle [\mathtt {oppt}] \rangle \varphi \\ \text{(reduction } \text{ of } \text{ second-order } \text{ opportunity) } &{} \langle [\mathtt {oppt}] \rangle \langle [\mathtt {oppt}] \rangle \varphi \rightarrow \langle [\mathtt {oppt}] \rangle \varphi \\ \text{(reduction } \text{ of } \text{ double } \text{ opportunity } \text{ negation) } &{} \lnot \langle [\mathtt {oppt}] \rangle \lnot \langle [\mathtt {oppt}] \rangle \varphi \rightarrow \langle [\mathtt {oppt}] \rangle \varphi \\ \text{(opportunities } \text{ induce } \text{ branching) } &{} \langle [\mathtt {oppt}] \rangle [\mathtt {X}] \varphi \rightarrow \langle \mathtt {H} \rangle [\mathtt {X}] [\mathtt {H}] \varphi \\ &{} \\ \text{ Power-Opportunity } \text{ interactions } &{} \\ \text{(opportunity-power } \text{ reduction) } &{} \langle [\mathtt {oppt}] \rangle \langle [ag \; \mathtt {pwr}] \rangle \varphi \leftrightarrow \langle [ag \; \mathtt {pwr}] \rangle \varphi \\ \text{(power-opportunity } \text{ reduction) } &{} \langle [ag \; \mathtt {pwr}] \rangle \langle [\mathtt {oppt}] \rangle \varphi \leftrightarrow \langle [\mathtt {oppt}] \rangle \varphi \\ \end{array} \end{aligned}$$

The above system is sound relative to the logic defined by the semantics, but we do not know if it is a complete characterisation. Let us state this in the following way.

### Proposition 4.1

(Soundness) $$Logic(\textsf {NBTS.PO}\text{-frames }) \supseteq \mathsf {NBTS.PO}_{Hilb}$$

Proofs are provided in the appendix.

### Open question 4.1

(Completeness) $$Logic(\textsf {NBTS.PO}\text{-frames }) {\mathop {\subseteq }\limits ^{?}} \mathsf {NBTS.PO}_{Hilb}$$

A proof strategy for completeness that could be employed here is to simulate the power and opportunity modalities in normal modal logic (Gasquet and Herzig 1993; Kracht and Wolter 1999). One meaningful way in which this could be done is by introducing normal modal logic S5 operators $[ag \; \mathtt {pwr}]$ and $[\mathtt {oppt}]$. Then we can define $\langle [ag \; \mathtt {pwr}] \rangle \varphi \equiv _{def} \langle H \rangle [ag \; \mathtt {pwr}] \varphi$ and $\langle [\mathtt {oppt}] \rangle \varphi \equiv _{def} \langle H \rangle [\mathtt {oppt}] \varphi$. It is not difficult to see that in this way we can build a normal modal logic equivalent of the above logic. For this equivalent logic, we could then aim for a normal modal logic completeness proof. One of the complications for completeness is exemplified by the ‘location independence of power’ axiom $\langle \mathtt {L} \rangle \langle [ag \; \mathtt {pwr}] \rangle \varphi \leftrightarrow \langle [ag \; \mathtt {pwr}] \rangle \langle \mathtt {L} \rangle \varphi$. The axiom expresses commutativity between the normal modal operator $[\mathtt {L}]$ and the non-normal operator $\langle [ag \; \mathtt {pwr}] \rangle$. Its validity is easy to assess. But, its translation in the above suggested way yields a non-Sahlqvist formula. Furthermore, the commutativity points to a product-like interaction between a normal and a non-normal modality, something that to our knowledge has received no attention in the mathematical modal logic literature (Kurucz 2007).

Instead of focussing on completeness, we discuss some salient valid schemas and non-valid schemas of the logic. We group related properties and issues together. We will use the symbol $\vdash$ for valid schemas derivable in the system, and $\not \vdash$ for schemas that are not derivable, and for which, due to soundness, semantical counterexamples exist.

### Counterfactual possibilities and the ‘reach’ of cross-historical reference

To assess properties like ‘opportunity for historical necessities’ $[\mathtt {H}] \varphi \rightarrow \langle [\mathtt {oppt}] \rangle \varphi$ it is important to realise that the modal quantifier $[\mathtt {H}]$ reaches over *all* histories emanating into the future from any given situation. These histories include some that interpret powers, some that interpret opportunities, some that interpret both, and we even allow those that interpret neither. Of the histories that interpret both, there are some that interpret *real* possibilities. The validity says that if a property $\varphi$ is true for all such histories, it must be a (trivial) opportunity. Validity of $[\mathtt {H}] \varphi \rightarrow \langle [ag \; \mathtt {pwr}] \rangle \varphi$ gives the same story for powers. The properties have negative variants in the form: $[\mathtt {H}] \varphi \rightarrow \lnot \langle [ag \; \mathtt {pwr}] \rangle \lnot \varphi$ and $[\mathtt {H}] \varphi \rightarrow \lnot \langle [\mathtt {oppt}] \rangle \lnot \varphi$.

The following properties are derivable from the ‘power for historical necessities/no power for historical impossibilities’ and ‘opportunity for historical necessities/no opportunity for historical impossibilities’ properties by substitution of $\top$.

$$\begin{aligned} \begin{array}{ll} {(power~for~logical~necessities) } &{} \vdash \langle [ag \; \mathtt {pwr}] \rangle \top \\ {(no~power~for~logical~impossibilities) } &{} \vdash \lnot \langle [ag \; \mathtt {pwr}] \rangle \bot \\ {(opportunity~for~logical~necessities) } &{} \vdash \langle [\mathtt {oppt}] \rangle \top \\ {(no~opportunity~for~logical~impossibilities) } &{} \vdash \lnot \langle [\mathtt {oppt}] \rangle \bot \\ \end{array} \end{aligned}$$

Note that by performing the substitution we detach a purely logical property from a property that carries both logical and physical information. And this is correct: logical invariants (in the sense of Tarski (1986)) are also invariant over time and space.

### Second-order phenomena, idempotency and immutability

$$\begin{aligned} \begin{array}{ll} {(idempotency~of~power) } &{} \vdash \langle [ag \; \mathtt {pwr}] \rangle \langle [ag \; \mathtt {pwr}] \rangle \varphi \leftrightarrow \langle [ag \; \mathtt {pwr}] \rangle \varphi \\ {(immutability~of~power) } &{} \vdash \lnot \langle [ag \; \mathtt {pwr}] \rangle \lnot \langle [ag \; \mathtt {pwr}] \rangle \varphi \leftrightarrow \langle [ag \; \mathtt {pwr}] \rangle \varphi \\ {(idempotency~of~opportunity) } &{} \vdash \langle [\mathtt {oppt}] \rangle \langle [\mathtt {oppt}] \rangle \varphi \leftrightarrow \langle [\mathtt {oppt}] \rangle \varphi \\ {(immutability~of~opportunity) } &{} \vdash \lnot \langle [\mathtt {oppt}] \rangle \lnot \langle [\mathtt {oppt}] \rangle \varphi \leftrightarrow \langle [\mathtt {oppt}] \rangle \varphi \\ \end{array} \end{aligned}$$

The properties of idempotency and immutability, that are easily derivable in the Hilbert system, express an important aspect of our modelling of power: there are no second-order powers. Agents have a power for something, or they do not have a power for that something. They do not have, in a non-trivial sense, the power to have a power, as this is just the same thing as having that power, witness the property of idempotency. And they do not have, in a non-trivial sense, the power not to have a power they have, as this would conflict with immutability. So agents have no (instantaneous) powers over their powers; in any situation there is exactly one specific unambiguously demarcated set of powers (which indeed is directly visible in the formal semantics in the form of the set $\mathcal {P}(m,ag)$).

Mayr (2011) mentions as an example of a second order power “to become brittle when heated” (the first order power is here “having the power to break”). But as we mentioned before, this assumes a more general interpretation of powers than ours. Here we strictly talk about *agentive* powers. However, it seems that the property we formulated for agentive powers could make sense for powers in general. It seems defendable to claim that in cases like “to become brittle when heated” the only relevant property is that the material (object) has the power to break and that we can eliminate all talk of brittleness: the object has the power to break, which is manifested if it is both heated and hit.

### Interactions between power and opportunity

Two properties listed among the axioms in the Hilbert system are the identification of the opportunity for power with plain power: $\langle [\mathtt {oppt}] \rangle \langle [ag \; \mathtt {pwr}] \rangle \varphi \leftrightarrow \langle [ag \; \mathtt {pwr}] \rangle \varphi$ and the identification of power for opportunity with plain opportunity: $\langle [ag \; \mathtt {pwr}] \rangle \langle [\mathtt {oppt}] \rangle \varphi \leftrightarrow \langle [\mathtt {oppt}] \rangle \varphi$. These properties follow from the fact that powers and opportunities each only select subsets of histories linked to the moments relative to which one evaluates truth. This eliminates rather complex possible concepts such as the opportunity for a power and the power for an opportunity relative to a *single* moment of evaluation. Note that concepts like ‘the power *to obtain* an opportunity’ may make sense though, if we consider agentive *movement* (we will discuss this separately below). However, in case of instantaneous realisations at one and the same moment of evaluation, it is correct to identify nestings of opportunity and power operators with the last operator used, as is reflected by the opportunity-power and power-opportunity reduction properties of Definition 4.4. From this it follows that the following are non-valid schemas saying that we do not have commutativity for the two non-normal operators.

$$\begin{aligned} \begin{array}{ll} {(no~opportunity-power~commutativity) } &{} \not \vdash \langle [\mathtt {oppt}] \rangle \langle [ag \; \mathtt {pwr}] \rangle \varphi \rightarrow \langle [ag \; \mathtt {pwr}] \rangle \langle [\mathtt {oppt}] \rangle \varphi \\ {(no~power-opportunity~commutativity) } &{} \not \vdash \langle [\mathtt {oppt}] \rangle \langle [ag \; \mathtt {pwr}] \rangle \varphi \leftarrow \langle [ag \; \mathtt {pwr}] \rangle \langle [\mathtt {oppt}] \rangle \varphi \\ \end{array} \end{aligned}$$

The counter models proving these invalidities are easy to construct, because for a given situation $\langle l, m, h \rangle$ powers and opportunities are entirely independent.

### Moving agents

One particularly interesting issue concerning interactions between power and opportunities is the problem of ‘agent movement’. Agents have a position somewhere in time and space. At such a point the agent has powers (that it would also have at other places in space) and it faces some opportunities (that are, or can be, specific for that situation). However, we can imagine that an agent lacking a certain opportunity might have the power and opportunity to *move* itself to a location where it *does* have that opportunity. Movement always takes place in time and space. And it cannot be infinitely fast. Should we then have logical properties in our system corresponding to the constraints imposed by the limitations of movement? We believe we should not. We already pointed to the fact that our object language ‘hides’ the locations of agents in the sense that they are not needed for the interpretation of the object level language operators. So, our language is not meant to be able to express such constraints. Movement couples the location dimension and the time dimensions. But in our language we can only talk about these dimensions in terms of general modal quantifiers; accounting for constraints reflecting the physical limitations of moving agents requires a stronger language, like the mathematical languages used by physicists.

### The dynamics of power and opportunity

A natural question to ask is how agentive powers and opportunities develop over time. It is clear that we do not want to put too much logical constraints on that. Opportunities change over time, because the world changes over time. And powers of agents change over time, because agents may gain or loose powers. However, for powers and opportunities that refer to future (next) properties, we do get a logical constraint. It is a quite subtle one and we explain it in terms of opportunities. If at a certain location there is an opportunity for an effect in the future, then along the histories leading into that particular future at that particular point in the future, there is an immediate (but trivial) opportunity for that effect.^28^ This is in accordance with the following properties following in the Hilbert system.

$$\begin{aligned} \begin{array}{ll} {(power\,time\,shift) } &{} \vdash \langle [ag \; \mathtt {pwr}] \rangle [\mathtt {X}] \varphi \rightarrow \langle \mathtt {H} \rangle [\mathtt {X}] \langle [ag \; \mathtt {pwr}] \rangle \varphi \\ {(opportunity\,time\,shift) } &{} \vdash \langle [\mathtt {oppt}] \rangle [\mathtt {X}] \varphi \rightarrow \langle \mathtt {H} \rangle [\mathtt {X}] \langle [\mathtt {oppt}] \rangle \varphi \\ \end{array} \end{aligned}$$

These are direct consequences of the ‘powers induce branching’ and ‘opportunities induce branching’ axioms in combination with the ‘power/opportunity for historical necessities’ axioms. One might wonder how then, with these logical constraints, agents obtain new powers and opportunities and lose others. The answer is: by being effective for some effects and not for others (something we will discuss in the next section). By being effective for a certain proposition, agents ensure that the ‘next’ histories are a subset of the current ones, and in the next state a new division of powers and opportunities in terms of this subset of histories becomes relevant. In this way, powers and opportunities change as time evolves.

### Independence of powers

We already explained that opportunities, since they belong to locations, are not independent, but hang together in a non-logical way determined by the physical constellation of the world. However, what about the powers of separate agents? Do they interfere with each other? Are there dependencies between them? Actually, in the system we put forward here there is such dependence and interference, but it goes via the notion of effectivity, discussed in the upcoming Sect. 5. Effectivity will be defined as requiring both power and opportunity. And for effectivity we will define an axiom capturing, for instance, the fact that it cannot be the case that at the same moment and location one agent effectively opens a door while another agent effectively closes it. But, in examples like these it does *not* follow that if one agent has the power to open the door, the other cannot have the power to close it; it can be that this other agent *has* the power, but not the opportunity. So, the logic dependence on the level of effectivity does not induce a logic dependence of powers, because this is prevented by the non-logical physically contingent way in which opportunities hang together across space. There is no systematic logic interaction at the level of powers; interactions between agents and between agents and their environment take place at the level of effectivity, and that is how it should be, we believe. This links up neatly with one of our core beliefs, which is that agentive powers are constitutive of what it is to be an agent. And being an agent or not should not depend on the environment or the interaction with other agents.

### Group powers and opportunities

A closely related question is whether powers and opportunities of agents can be combined into powers and opportunities of groups of agents. Let us first note that we do not have groups in the language here. Several related formalisms like Coalition Logic (Pauly 2002) and Alternating Time Temporal logic (Alur et al. 2002), do. The central property in these formalisms, called ‘super-additivity’, allows that the effectivity of groups possibly exceeds the effectivity of its members. Should we allow a similar view for group powers and group opportunities? Our current position is that the powers of a group are nothing more than the union of the powers of the individuals in the group. We believe that powers are what make agents into individual agents, and groups are not agents in that same sense. If that is correct, for powers we should trade in Coalition Logic’s super-additivity axiom for the (logically) stronger ‘additivity’ axiom. But how then should we think about the role of opportunities? As we saw, opportunities are not properties of agents, but of the locations these agents apply their powers to. This would not change for groups of agents: the powers of groups combine with the opportunities of locations to form possibilities for these groups to be effective. But, we could maybe add-in an extra interaction here, and it is one that could explain how on the level of effectivity we switch from additivity to super-additivity. This interaction consists in the possibility that agents might use characteristics of other agents in their environment as *opportunities* (which is inspired by the ideas of Bratman on shared agency; Bratman 2014). However, all this is currently not accommodated by our theory, and we leave the issue of powers and opportunities of groups for future research.

## Effectivity

We will now add effectivity to the picture. An agent can be effective in a situation if it has the appropriate power and the situation ‘grants’ him the opportunity, that is, the situational environment (objects, forces, orientations, etc.) does not constrain it in exercising its power. A power that can be exercised in a situation with the appropriate opportunities leads to a real choice. A real choice is a *set* of real possibilities, and an agent having that choice has the possibility to be effective by constraining the future possible courses of events to those demarcated by the choice.

Let us again make these intuitions formal. We introduce an effective *stit* operator to model the concept of effectivity.

### Definition 5.1

Given a countable set of propositions *P* and $p \in P$, and given a finite set *Ags* of agent names, and $ag \in Ags$, the formal language $\mathcal L_{\textsf {ESTIT}}$ is:

$$\begin{aligned} \begin{array}{lcl} \varphi &{} := &{} p \mid \lnot \varphi \mid \varphi \wedge \varphi \mid [\mathtt {L}] \varphi \mid [\mathtt {X}] \varphi \mid [\mathtt {H}] \varphi \mid \langle [ag \; \mathtt {pwr}] \rangle \varphi \mid \langle [\mathtt {oppt}] \rangle \varphi \mid [ag \; \mathtt {Estit}] \varphi \\ \end{array} \end{aligned}$$

The effective *stit* operator $[ag \; \mathtt {Estit}]$ has the following reading.

- $[ag \; \mathtt {Estit}] \varphi$ reads “relative to the situation of evaluation, agent *ag* effectively sees to it that $\varphi$”.

For convenience we also provide the reading of the dual.

- $\langle ag \; \mathtt {Estit} \rangle \varphi$ reads “relative to the situation of evaluation, agent *ag* effectively allows for $\varphi$ to occur”.

The dual accurately captures modes of acting that play a significant role in the moral or legal evaluation of acts (one of the subject matters of deontic logic; Gabbay et al. 2013); agents can be to blame for things they allowed to happen (and could have prevented^29^).

We will *not* assume that an agent can only have an effect in the situation of evaluation if it is actually *present* at the position determined by that situation (remember that situations have three components: a location, a moment and a history). By choosing, an agent affects the entire space it inhabites since branching is *not* a local phenomenon in our theory (see Sects. 1 and 2); in terms of the histories selected by an agent that is present *somewhere* in space exercising its choice, a choice is uniform across space.

Before giving the formal definition of the effectivity frames, let us introduce the reader a bit more to *stit*-stuctures in general. A *stit*-structure imposes a structure of moment-based choices on a branching time structure. In such structures it makes less sense to ask “does the agent only have one choice to make at any moment?”. The answer is either yes or no, depending on how one individuates the act to be done by the agent. *Yes*, one choice can only be done in the sense that one subset of histories is carved out from all the possible temporal continuations of the world; in a non-deterministic world it simply makes no sense to assume that agents can do anything else than that. *No*, this one set of histories carved out by what an agent choses to do may be described as doing many things at the same time; eating ones meal, thinking about logic, looking at ones partner, listening to music, each of which can also be seen as a separate choice, either conscious or unconscious, either intentional or unintentional. The structures we define below always ‘look’ at a set of histories necessitated by an agent’s choice (in the first sense described above) from the perspective of one such history in the set. We will call the function that gives the alternative histories relative to a situation $\langle l, m, h \rangle$ an ‘effectivity function’, because it gives the choice that determines for which properties the agent is effective relative to the history *h*. This, of course, can be rather confusing for readers familiar with the Coalition Logic notion of effectivity function (Pauly 2002), which actually, as already explained, is similar to the functions we use to interpret powers and opportunities. To avoid confusion (somewhat) we will call the effectivity function we use below a ‘situational h-effectivity function’.

We extend the NBTS.PO-frames $\langle L, M, H, \mathcal {L}, \mathcal {X}, \mathcal {H}, \mathcal {P}, \mathcal {O} \rangle$ of Definition 4.2 to ESTIT-frames $\langle L, M, H, \mathcal {L}, \mathcal {X}, \mathcal {H}, \mathcal {P}, \mathcal {O}, \mathcal {E} \rangle$ that include a function $\mathcal {E}$ encoding effectivity.

### Definition 5.2

An ESTIT-frame is a tuple $\langle L, M, H, \mathcal {L}, \mathcal {X}, \mathcal {H}, \mathcal {P}, \mathcal {O}, \mathcal {E} \rangle$ such that:

1.: $\langle L, M, H, \mathcal {L}, \mathcal {X}, \mathcal {H}, \mathcal {P}, \mathcal {O} \rangle$ is an NBTS.PO-frame.
2.: $\mathcal {E}: L \times M \times H \times Ags \mapsto 2^{H}$ is a situational h-effectivity function yielding for an agent *ag* the set of histories allowed by the choice exercised by the agent relative to a location *l*, moment *m* and history *h*. The agent *ag* need not be present at location *l*. If agents are ineffective relative to a situation, the effectivity function assigns the empty set. We have the following constraints on situational h-effectivity functions^30^:
  a.: **no effectivity in incoherent situations:** if $m \not \in h$ then $\mathcal {E}(l, m, h, ag) = \emptyset$
  b.: **conditional success:** if $\mathcal {E}(l, m, h, ag) \not = \emptyset$ then $h \in \mathcal {E}(l, m, h, ag)$
  c.: **moment specificity of effectivity:** if $h' \in \mathcal {E}(l, m, h, ag)$ then $m \in h'$
  d.: **effectivity requires power:** if $\mathcal {E}(l, m, h, ag) \not = \emptyset$ then $\mathcal {E}(l, m, h, ag) \in \mathcal {P}(m,ag)$
  e.: **effectivity requires opportunity:** if $\mathcal {E}(l, m, h, ag) \not = \emptyset$ then $\mathcal {E}(l, m, h, ag) \in \mathcal {O}(l,m)$
  f.: **local real possibility:** there is an $h \in H$ such that $\mathcal {E}(l, m, h, ag) \not = \emptyset$
  g.: **shared space dependence:** if for $0 < i \le \left| {Ags} \right|$, $\mathcal {E}(l_i, m, h_i, ag_i) \not = \emptyset$ then $\bigcap \nolimits _{0 < i \le \left| {Ags} \right| } \mathcal {E}(l_i, m, h_i, ag_i) \not = \emptyset$

Property 2.a. says that for incoherent situations $\langle l, m, h \rangle$ where the moment *m* is not an element of the history *h*, the effectivity function specifies the empty effect. As we already mentioned, such incoherent situations will never ‘occur’, however, we add this condition to make the function $\mathcal {E}$ total. This constraint does not in any way affect the properties of the logic.

Property 2.b. says that if we evaluate against a real history, that history is in our effectivity set. This is a conditional version of classical *stit*’s success property: effectivity implies succes.

Property 2.c. says that choices as defined by the effectivity function are only effective for the moment they are exercised at.

Properties 2.d. and 2.e. say that effectivity requires both power and opportunity. Note that the distinction between effectivity, power and opportunity induces different kinds of histories. In particular, there are histories that are *not* part of the effectivity function of an agent. This is a main departure from standard *stit* theory, where histories are always concrete possibilities. But in our current theory there are also histories that only give semantics to an agent’s powers or to opportunities of locations without being elements of an effectivity function. We call such histories ‘counter factual possibilities’.

Also note that effectivity possibly takes more than only power and opportunity. We do not exclude that additional concepts are needed to get to a sufficient condition for being effective. One such concept could be ‘knowing how’ (Ryle 1946).

At this point it is opportune to point to a constraint we did *not* explicitly state: h. **no choice between undivided histories:** if $h' \in \mathcal {E}(l, m, h, ag)$ and $h'' \in H$ and $m \in h''$ and $next_{h'}(m) = next_{h''}(m)$, then $h'' \in \mathcal {E}(l, m, h, ag)$. The reason we did not state this constraint is that it already follows from conditions 2.d. and 2.e. together with the **powers induce branching** and **opportunities induce branching** conditions in Definition 4.2. Because effectivity sets (sets of histories pointed to by $\mathcal {E}(l, m, h, ag)$ for different *h*) are also power sets and opportunity sets, and because the ‘undivided histories’ condition is logically equivalent to the ‘induced branching’ properties, there is no need to explicitly state it again (see proof in the appendix for the soundness of the ‘no choice between undivided histories’ axiom in Definition 5.4).

Property 2.f. captures a basic assumption: that for any moment and location, there is a real future that is admitted by any agent. The alternative would be incoherent: it would mean that there could be moments and locations where agents could effectively see to it that history comes to a hold. One could maybe argue that this could be allowed as a kind of *subjective* possibility, for instance to model that for a certain agent history would come to a hold (the agent is no longer part of the system; it dies and its powers cease to exist,^31^ not their opportunities), but then we would enter even more uncharted territory.

Property 2.g. is the property we have to say about most. The property is a spatial generalisation of what in the *stit* literature is called ‘independence of agency’. But, that name is a mistake, we believe, and it is one that has carried a long way. First note that the property is about non-emptyness of intersections: it says that if somewhere in space there is an agent performing a real choice (it is effective for something), and somewhere else in space there is another agent also performing a real choice, then the intersection of their choices (in terms of admitted histories) is non-empty. Now one can have different views on what non-emptyness means here. Our view is that non-emptiness of intersections of choices of spatially separated agents represents that one agent can *influence* the other in the sense that one agent can rule out possible futures for the other. If an agent throws a stone in a river, it thereby changes the world common to all agents and possibly influences, in some inexplicable and unknown way, the possible futures of those other agents. The influence is there, because the throwing of the stone rules out some of the cross spatial joint futures that were possible before the stone throwing. If we would allow intersections to be empty, we could not conclude to this dependency. If two spatially separated agents can perform their choices entirely *independently* thereby reaching a new situation where they no longer share a joint cross spatial history, apparently they have started to live in spatially entirely separated universes; they can no longer do actions that at some point in the future might have a joint consequence.^32^

Now, within this view, there is a case to be made for the position that the further away two agents are from each other, the more *independent* they become. Intuitively this is right. And the reason is just that the further away agents are, for all practical purposes, the more they start to live in separated worlds. The further away they are, the less likely it will become their actions will ever interfere. But gradients is not what we are studying here. Our logic is not about likelihoods of interaction or grades of dependence. In our idealised logical sense agents that live in the same world, no matter how far they are spatially removed from each other, are dependent; they live in the same space which means that for whatever actions they perform, ultimately there might be joint consequences within that shared space.

Let us now briefly look at the other view. The traditional narrative behind non-emptyness of choice intersections in *stit* logic is quite different. In a sense it is exactly the opposite. It goes something like this. The choices of one agent cannot be influenced by the choices of another agent, because whatever choice one agent performs, any choice of some other agent is still possible because of the non-emptyness of intersections. Note how this narrative leads to exactly the opposite point of view: if intersections of choices are non-empty, the agents are **in***dependent*, because a choice by one agent does *not affect* the repertoire of choices open to other agents. We believe our narrative above, with a clear role of the spatial dimension of agency, is much more appealing, which is why we reject the name ‘independence of agency’ for the most central formal property of *stit* logic’s. The generalisation to a situational context involving both time and space shows us that the property expresses *dependence* rather than independence.^33^ This is especially clear if one realises that in the traditional view, we cannot develop a story of agents ‘in practice’ becoming *more* independent the further they become spatially separated, as we did above for our interpretation.

We end this discussion point with what Belnap, in an early article (Belnap 1991), has to say about the non-emptyness of intersections. Here is what Belnap writes:

When considering multiple agents, I postulate for each moment that for each way of selecting one possible choice for each agent from among his or her set of choices, the intersection of all the possible choices selected must contain at least one history (Something happens).

Note that this quote does not talk about independence or dependence, but more neutrally about intersections in relation to ‘something happens’. Belnap’s description concerns the classical *stit* picture that does not consider a spatial dimension nor counterfactual possibilities related to powers and opportunities, as we do here. But, translated to our setting, the property is directly linked to our dependency property, and to our property 2.f concerning local real possibility. Note however that ‘something happens’ is maybe not a very good name for the property described in the quote; it may suggest that one of the histories in the non-empty intersection is ‘most real’ in the sense that it is the actual thing that is happening. That is a wrong idea, also heavily attacked by Belnap himself (see his discussion on the ‘thin red line’; Belnap and Green 1994). But if we adopt Belnap’s terminology here, we could say that in our semantics we do not only have temporal continuations along which ‘something happens’, but also non-real continuations where we could say that *nothing* happens,^34^ or something ‘could have happened’ if the right opportunities and/or powers would have been in place.

Let us proceed with the truth condition for the effectivity operator. We evaluate truth with respect to situations built from a dimension of locations, a dimension of moments and a dimension of histories.

### Definition 5.3

Relative to a model $\langle L, M, H, \mathcal {L}, \mathcal {X}, \mathcal {H}, \mathcal {P}, \mathcal {O}, \mathcal {E}, V \rangle$, truth $\mathcal \langle l, m, h \rangle \!\models \varphi$ of a formula $\varphi$ in a space–time situation $\langle l, m, h \rangle$ with $m \in h$ is defined as^35^:

$$\begin{aligned} \begin{array}{lll} \langle l, m, h \rangle \models [ag \; \mathtt {Estit}] \varphi &{} \Leftrightarrow &{} h' \in \mathcal {E}(l, m, h,ag) \text{ implies } \mathcal \langle l, m, h' \rangle \models \varphi \\ \end{array} \end{aligned}$$

Satisfiability, validity on a frame and general validity are defined as usual.

If $\mathcal {E}(l, m, h,ag) = \emptyset$ we have that $\langle l, m, h \rangle \models \lnot \langle ag \; \mathtt {Estit} \rangle \top$. This says that effectivity allowing for any consistent logical property is not possible along the history *h*; the agent can only be effective along the history on penalty of reaching a logically inconsistent state. This is another way to say that such effectivity is not possible (physically).

Note how all our operators $[ag \; \mathtt {Estit}], \langle [ag \; \mathtt {pwr}] \rangle$ and $\langle [\mathtt {oppt}] \rangle$ are interpreted as carrying truths about the set of histories that can be reached by an *instantaneous* ‘jump’ relative to the same moment and choice.

### Definition 5.4

The following axiom schemas, in combination with a standard axiomatization for propositional logic, and the standard rules *(*like necessitation*)* for the normal modal operators ‘$[\;]$’, and the non-normal operators ‘$\langle [\; ] \rangle$’ define the Hilbert system $\mathsf {ESTIT}_{Hilb}$:

$$\begin{aligned} \begin{array}{ll} \text{(agglomeration } \text{ and } \text{ monotonicity) } &{} [ag \; \mathtt {Estit}] \varphi \wedge [ag \; \mathtt {Estit}] \psi \leftrightarrow [ag \; \mathtt {Estit}] (\varphi \wedge \psi ) \\ \text{(transitivity } \text{ and } \text{ Euclidicity) } &{} \text{ axioms } \text{4 } \text{ and } \text{5 } \text{ for } \text{ each } [ag \; \mathtt {Estit}] \\ \text{(conditional } \text{ success) } &{} \langle ag \; \mathtt {Estit} \rangle \top \rightarrow ([ag \; \mathtt {Estit}] \varphi \rightarrow \varphi ) \\ \text{(effectivity } \text{ for } \text{ historical } \text{ necessities) } &{} [\mathtt {H}] \varphi \rightarrow [ag \; \mathtt {Estit}] \varphi \\ \text{(effectivity } \text{ requires } \text{ power } \text{ and } \text{ opportunity) } &{} \langle \mathtt {H} \rangle [ag \; \mathtt {Estit}] \varphi \rightarrow \langle [ag \; \mathtt {pwr}] \rangle \varphi \wedge \langle [\mathtt {oppt}] \rangle \varphi \\ \text{(no } \text{ choice } \text{ between } \text{ undivided } \text{ histories) } &{} \langle ag \; \mathtt {Estit} \rangle \top \rightarrow ([ag \; \mathtt {Estit}] [\mathtt {X}] \varphi \rightarrow [\mathtt {X}] [\mathtt {H}] \varphi ) \\ \text{(local } \text{ real } \text{ possibility) } &{} \langle \mathtt {H} \rangle \langle ag \; \mathtt {Estit} \rangle \top \\ \text{(shared } \text{ space } \text{ dependence) } &{} \bigwedge \limits _{ag_i \in Ags} \langle \mathtt {H} \rangle \langle \mathtt {L} \rangle (\langle ag \; \mathtt {Estit} \rangle \top \wedge [ag_i \; \mathtt {Estit}] \varphi _i) \rightarrow \\ &{} \langle \mathtt {H} \rangle \bigwedge \limits _{ag_i \in Ags} \langle \mathtt {L} \rangle (\langle ag \; \mathtt {Estit} \rangle \top \wedge [ag_i \; \mathtt {Estit}] \varphi _i) \\ \end{array} \end{aligned}$$

The above system is sound relative to the logic defined by the semantics, but, as for the fragment involving only powers and opportunities in Sect. 4, we do not know if it is a complete characterisation. We state this more precisely, as follows:

### Proposition 5.1

(Soundness) $$Logic(\textsf {ESTIT}\text{-frames }) \supseteq \mathsf {ESTIT}_{Hilb}$$

For the proofs, see the appendix.

### Open question 5.1

(Completeness) $$Logic(\textsf {ESTIT}\text{-frames }) {\mathop {\subseteq }\limits ^{?}} \mathsf {ESTIT}_{Hilb}$$

As in Sect. 4 for powers and opportunities, let us discuss salient properties and non-properties of this semantics in order to sharpen our intuitions and assess its appropriateness. We will again use $\vdash$ for valid schemas derivable in the system, and $\not \vdash$ for schemas for which counterexamples exist.

### Something happens (better: global real possibility)

We can capture Belnap’s ‘something happens’ intuition (see our earlier discussion) in a derivable property that we call ‘global real possibility’ (recall that we did not agree with Belnap’s name for the property). In the following schema we have that $n = \left| {Ags} \right|$.

$$\begin{aligned} \begin{array}{ll} {(global\;real\;possibility) } &{} \vdash \langle \mathtt {H} \rangle (\langle \mathtt {L} \rangle \langle ag_1 \; \mathtt {Estit} \rangle \top \wedge \ldots \wedge \langle \mathtt {L} \rangle \langle ag_n \; \mathtt {Estit} \rangle \top ) \\ \end{array} \end{aligned}$$

The property is derivable from the properties ‘local real possibility’ and ‘shared space dependence’. It says there is always some future admitted by the agents that are in the spatio-temporal space our location of evaluation is part of. In terms of the properties it derives from, we can say that local real possibilities can always be combined into global real possibilities due to the property of different local possibilities always being related to each other in a shared space. Note that in our semantics this property also holds true for situations that are ‘reached’ through non-real histories. We believe that is exactly right; also in counter-factual situations we apply the logical constraints that hold for ‘real’ situations.

### Interactions between effects and space

If an agent is effective, it is, in a logical sense, effective everywhere in space, because the histories admitted by its choices are global and stretch out over the whole of space. That is the Newtonian picture we sketched before. However, from that it does not follow that we have a property like $\langle ag \; \mathtt {Estit}\rangle \top \rightarrow [\mathtt {L}] \langle ag \; \mathtt {Estit}\rangle \top$ that would express something like ‘cross-spatial possibility’. Relative to a *fixed* history *h*, varying over the spatial coordinate *l*, opportunities change, and thus, also the possibility for an agent to be effective. Two related schemas concerning effects and space that do not hold are the following (to keep the schemas simple we here consider systems with 2 agents only).

$$\begin{aligned} \begin{array}{ll} {(spatial\;separation\;of\;effects) } &{} \not \vdash \langle \mathtt {L} \rangle [ag \; \mathtt {Estit}] \varphi \wedge \langle \mathtt {L} \rangle [ag \; \mathtt {Estit}] \psi \rightarrow \\ &{} \langle \mathtt {L} \rangle ([ag \; \mathtt {Estit}] \varphi \wedge [ag \; \mathtt {Estit}] \psi ) \\ {(location\;dependence) } &{} \not \vdash \langle \mathtt {L} \rangle [ag_1 \; \mathtt {Estit}] \varphi \wedge \langle \mathtt {L} \rangle [ag_2 \; \mathtt {Estit}] \psi \rightarrow \\ &{} \langle \mathtt {L} \rangle ([ag_1 \; \mathtt {Estit}] \varphi \wedge [ag_2 \; \mathtt {Estit}] \psi ) \\ \end{array} \end{aligned}$$

The schemas are not valid, because effectivity rests on opportunity, which depends on the location. Counter models can thus easily be constructed on the basis of different opportunities for different locations that cannot be made to hold jointly for one location.

### Unreal histories and partiality of effectivity

Our semantics admits different kinds of histories. We already pointed to the difference between real histories (for which $\mathcal {E}(l, m, h, ag) \not = \emptyset$) and unreal (counterfactual) histories (for which $\mathcal {E}(l, m, h, ag) = \emptyset$). Unreal histories are ones along which agents are not effective. The existence of such histories leads to the absence of seriality for effectivity.

$$\begin{aligned} \begin{array}{ll} {(no\;seriality\;for\;effectivity) } &{} \not \vdash \lnot [ag \; \mathtt {Estit}] \bot \\ \end{array} \end{aligned}$$

As a consequence, we also do not have unconditional success for effectivity: $\not \vdash [ag \; \mathtt {Estit}] \varphi \rightarrow \varphi$, which is why we added the condition to the success schema in the Hilbert system.

For exactly the same reason (unreal histories) also the following scheme does not hold (note that for power and opportunity we had similar schemas that *did* hold).

$$\begin{aligned} \begin{array}{ll} {(no\;absence\;of\;effectivity\;for\;historical\;impossibilities) } &{} \not \vdash [\mathtt {H}] \varphi \rightarrow \lnot [ag \; \mathtt {Estit}] \lnot \varphi \\ \end{array} \end{aligned}$$

Clearly we can also have histories that are ‘partially’ real. A history *h* can be real up to the moment *m* where $\mathcal {E}(l, m, h, ag) = \emptyset$. And an unreal history can come back real again at a moment $m'$ where $\mathcal {E}(l, m', h, ag) \not = \emptyset$. And histories can also be partially real in another sense: real for one agent but not for another. Along such histories some agents are effective, while others are not. So the counterfactuality of such histories is due to the fact that some agents in the situation considered do not have the right powers or opportunities to be effective. One could add as a constraint to the logic that we want to rule out such partial effectiveness. But we do not. Since we do not, we have the following property.

$$\begin{aligned} \begin{array}{ll} {(partiality\;of\;effectivity) } &{} \not \vdash \lnot \langle \mathtt {H} \rangle (\langle ag_1 \; \mathtt {Estit} \rangle \top \wedge [ag_2 \; \mathtt {Estit}] \bot ) \\ \end{array} \end{aligned}$$

### The relation to the classical axiom for independence

The classical axiom of independence for stit-logics is as follows (for ease of exposition, we only consider two-agent systems in this discussion).

$$\begin{aligned} \begin{array}{ll} \text {(classical 2-agent `independence')} &{} \langle \mathtt {H} \rangle [ag_1 \; \mathtt {Estit}] \varphi \wedge \langle \mathtt {H} \rangle [ag_2 \; \mathtt {Estit}] \psi \rightarrow \\ &{} \langle \mathtt {H} \rangle ([ag_1 \; \mathtt {Estit}] \varphi \wedge [ag_2 \; \mathtt {Estit}] \psi ) \\ \end{array} \end{aligned}$$

The axiom and the associated constraint on frames are justifiable if they talk about a world consisting of moments and (branching) histories, but no locations. It is a natural question then if in the current setup, where locations are added, the classical axiom comes back if, for instance, we consider what is valid relative to one particular fixed location. For two-agent systems, our constraint for *shared space dependence* is: if $\mathcal {E}(l, m, h, ag_1) \not = \emptyset$ and $\mathcal {E}(l', m, h', ag_2) \not = \emptyset$ then $\mathcal {E}(l, m, h, ag_1) \cap \mathcal {E}(l', m, h', ag_2) \not = \emptyset$. Now, if we consider this constraint relative to one particular location, which means that $l = l'$, we get a corresponding schema that is close to the classical independence axiom.

$$\begin{aligned} \begin{array}{l} \text {(2-agent `shared space dependence')} \\ \langle \mathtt {H} \rangle (\langle ag_1 \; \mathtt {Estit} \rangle \top \wedge [ag_1\; \mathtt {Estit}] \varphi ) \wedge \langle \mathtt {H} \rangle (\langle ag_2 \; \mathtt {Estit} \rangle \top \wedge [ag_2 \; \mathtt {Estit}] \psi ) \rightarrow \\ \langle \mathtt {H} \rangle (\langle ag_1 \; \mathtt {Estit} \rangle \top \wedge [ag_1 \; \mathtt {Estit}] \varphi \wedge \langle ag_2 \; \mathtt {Estit} \rangle \top \wedge [ag_2 \; \mathtt {Estit}] \psi ) \\ \end{array} \end{aligned}$$

If the Hilbert system we gave is complete, this schema is derivable. But here we do not want to investigate that, we only want to point to what the schema means and how it can be justified that it closely resembles the classical axiom for independence (the only difference being the ‘possibility’ clauses of the form $\langle ag_i \; \mathtt {Estit} \rangle \top$).

One can easily be led to belief that actually there are obvious counterexamples falsifying this schema. Those are the examples where we substitute (logically) conflicting information for $\varphi$ and $\psi$. Take for instance the example where one agent, relative to a house at the current location, ensures that he will repaint it (let us say, represented by seeing to it that *p*), while another agent ensures that it will be burned down (implying and amounting to seeing to it that $\lnot p$). Clearly there is not one history admitting this, since the same house cannot be repainted and burned down at the same time. But this does not invalidate the above schema. It only says that since in this case the consequence is false, the antecedent also has to be false. So, we get the property $\lnot (\langle \mathtt {H} \rangle (\langle ag_1 \; \mathtt {Estit} \rangle \top \wedge [ag_1\; \mathtt {Estit}] \varphi ) \wedge \langle \mathtt {H} \rangle (\langle ag_2 \; \mathtt {Estit} \rangle \top \wedge [ag_2 \; \mathtt {Estit}] \psi ))$, by which it follows that if at some place of evaluation it is possible for some agent to make *p* true (the painting of the house), it is excluded that at that same place it is possible for some other agent to make $\lnot p$ true (the burning of the house). These possibilities cannot coexist, because, they depend on each other: in case the house is indeed burned down by agent 1, it is not possible for agent 2 to repaint it. This emphasises again that despite its name, the classical axiom expresses *dependence* and not *independence*: the possibility for some agent to repaint the house *depends* on the house not being burned down by some other agent.^36^

Now let us investigate this same example in our spatially generalised logic. Assume that we still have that seeing to it that *p* means that a house is repainted and that $\lnot p$ stands for burning down a house. But now the seeing to it that *p* and the seeing to it that $\lnot p$ take place by different agents *at different locations*. Then, for this specific example, there is no dependence anymore: since the effectivity for *p* and for $\lnot p$ takes place at different places, apparently we are talking about different houses. And then there is no conflict and no dependence; one house is repainted, the other burned down.

But, if it is the case that in our generalised spatial setting, examples like these show that agents acting at different locations can be more *independent*, then why are we still referring to our axiom as “shared space *dependency*”? That is because even if two agents are far removed from each other, as long as they share the same physical space, in principle there can be cases of dependence or interference. In the burning down the house example this can be made clear in an admittedly somewhat contrived way. Assume that the second agent, the one with the arsonist tendencies, has the opportunity to employ a powerful bomb that can destroy the world. That would mean that no houses wherever located could be repainted, which could be associated with making the formula $[\mathtt {L}] \lnot p$ true.^37^ So, now again, this conflicts with *p* being true at some other location. Then, in this adapted example, we have dependence again; and the dependence is due to the fact that the agents inhabit the same space.

## Conclusion

In this article we are interested in logical theories of agency where choice performance is situated in time and space. We take Chellas’ stit logic (Chellas 1980) as the basis for our investigations and add a spatial dimension to it to come to a theory of *situated* agency.

The only other theory aiming at a theory of agency in time and space is Belnap’s. Unlike Belnap, we do not only study *semantic structures* of space, time and agency, but also add the space dimension to a logical *object language*. The differences with Belnap do not stop there. An important difference is that we depart from a classical Newtonian picture, while Belnap’s proposal for agency in time and space is based on his earlier work on relativistic branching space–times (BST, for short) (Belnap 1992). The theory of branching space–times is very intriguing as it couples a ‘true’ description of space and time, namely Einstein’s general relativity, with the crucial presupposition of *stit* theory and open future/indeterministic thinking: the reality of *branching* time. However, we have good reasons to stick to a Newtonian picture, as we explained. A third difference with Belnap is that we incorporate in our spatio-temporal *stit* logic how opportunities linked to ‘locations’ and ‘powers’ linked to agents, together may result in ‘real possibilities’ or ‘factual possibilities’ that may give rise to concrete instances of agents being effective. We managed to give a precise logical description of the relation between opportunity, power and effectivity. This precise logical description distinguishing between two different kinds of ‘non-real’ possibilities in the semantics. We can have counterfactual possibilities associated with opportunities at specific spatial locations that would have been real possibilities if an agent with the right powers would be present at that location, and we can have counterfactual possibilities associated with agents present at certain locations; agents having certain powers that would have been real possibilities if at that location the agent would have had the opportunity the effectuate its power.

The theory we have put forward provokes new questions that could not be asked before. An example of such a question is if it is possible to give a similar object language level analysis of Belnap’s theory. It is clear that some of the axioms central to our formal system, like ‘global clocks’ and ‘global branching’ should not hold for a language taking Belnap’s model theory as its interpretation. But it is unclear what we would get back in return, and how ideas about powers and opportunities could be added to Belnap’s view.

Admittedly, this article has several deficiencies. There are many open questions. Also the chosen approach and the design choices for the logic are open to reconsideration. For instance: would it not have been better to be less abstract about space? To make that question more concrete: if time comes with a discretisation (the next operator), why do we not do the same for space? And, should the language (and thus the structures) not be more concrete about the locations of agents? We can ask several more questions like these. This shows that we have only scratched the surface of the problem of formalising situated agency. Yet we believe our picture can be a starting point for new developments.

Maybe the most important contribution of the article is the picture it sketches of agency in time and space. To conclude we want to try to sketch that picture one more time. Every agent at any point in time and space can change the course of history of its universe. If an agent does so at some point in time and space, that agent also changes the course of history relative to all other points in space. In that *logical* sense effects on other locations are instantaneous. It follows that in a fundamental sense all space-inhabiting agents work together to determine the future of their spatial world. Branching is then a global phenomenon influenced by local choices of agents. These local choices, on their part, depend on agentive powers and local opportunities. All these dispositional properties together, in one huge Cartesian product, determine the possible real futures of the world.

## Appendix: Soundness proofs

### Proofs for proposition 4.1

$$\begin{aligned} \begin{array}{ll} {{\text {(moment determinacy of power)}}} &{} \langle [ag \; \mathtt {pwr}] \rangle \varphi \rightarrow [\mathtt {H}] \langle [ag \; \mathtt {pwr}] \rangle \varphi \\ \end{array} \end{aligned}$$

$\langle l, m, h \rangle \models \langle [ag \; \mathtt {pwr}] \rangle \varphi$ says that $\exists P \in \mathcal {P}(m,ag) \text{ such } \text{ that } \forall h' \in P, \langle l, m, h' \rangle \models \varphi$. Now, since the function $\mathcal {P}(m,ag)$ does not depend on histories, we directly have that for any $h''$ running through *m* it holds that $\langle l, m, h'' \rangle \models \langle [ag \; \mathtt {pwr}] \rangle \varphi$. But then $\langle l, m, h \rangle \models [\mathtt {H}] \langle [ag \; \mathtt {pwr}] \rangle \varphi$. $\square$

$$\begin{aligned} \begin{array}{ll} {{(power\;for\;historical\;necessities)}} &{} [\mathtt {H}] \varphi \rightarrow \langle [ag \; \mathtt {pwr}] \rangle \varphi \\ \end{array} \end{aligned}$$

From condition 2.a of Definition 4.2 we have that powers are subsets of histories relative to an agent *ag* running through a particular moment *m*. Because of condition 2.b of Definition 4.2 we know that $\exists P \in \mathcal {P}(m,ag)$. Now, if all situations based on the histories running through *m* satisfy $\varphi$, also the situations based on histories in *P* satisfy it.

$$\begin{aligned} \begin{array}{rr} {(no\;power\;for\;historical\;impossibilities) } &{} [\mathtt {H}] \varphi \rightarrow \lnot \langle [ag \; \mathtt {pwr}] \rangle \lnot \varphi \\ \end{array} \end{aligned}$$

It is convenient to prove the contraposition $\langle [ag \; \mathtt {pwr}] \rangle \lnot \varphi \rightarrow \lnot [\mathtt {H}] \varphi$. If $\langle l, m, h \rangle \models \langle [ag \; \mathtt {pwr}] \rangle \lnot \varphi$ then $\exists P \in \mathcal {P}(m,ag) \text{ such } \text{ that } \forall h' \in P, \langle l, m, h' \rangle \models \lnot \varphi$. Now condition 2.c of Definition 4.2 tells us that $P \not = \emptyset$. But then also $\exists h' \in P$ such that $\langle l, m, h' \rangle \models \lnot \varphi$. But then $\langle l, m, h \rangle \not \models [\mathtt {H}] \varphi$ and thus $\langle l, m, h \rangle \models \lnot [\mathtt {H}] \varphi$.

$$\begin{aligned} \begin{array}{rr} {(location\;independence\;of\;power) } &{} \langle \mathtt {L} \rangle \langle [ag \; \mathtt {pwr}] \rangle \varphi \leftrightarrow \langle [ag \; \mathtt {pwr}] \rangle \langle \mathtt {L} \rangle \varphi \\ \end{array} \end{aligned}$$

Directly from the fact that powers are location independent subsets of histories and the fact that the history dimension and the location dimension commute (def 3.5).

$$\begin{aligned} \begin{array}{rr} {(monotonicity\;of\;power) }&\langle [ag \; \mathtt {pwr}] \rangle (\varphi \wedge \psi ) \rightarrow \langle [ag \; \mathtt {pwr}] \rangle \varphi \end{array} \end{aligned}$$

Directly from the fact that powers are sets (of histories, relative to a moment and an agent). If $\varphi \wedge \psi$ holds for a set, $\varphi$ also does.

$$\begin{aligned} \begin{array}{rr} {(reduction\;of\;second-order\;power) } &{} \langle [ag \; \mathtt {pwr}] \rangle \langle [ag \; \mathtt {pwr}] \rangle \varphi \rightarrow \langle [ag \; \mathtt {pwr}] \rangle \varphi \\ \end{array} \end{aligned}$$

Consider a situation $\langle l, m, h \rangle$ relative to a model $\langle L, M, H, \mathcal {L}, \mathcal {X}, \mathcal {H}, \mathcal {P}, \mathcal {O}, V \rangle$ such that $\langle l, m, h \rangle \models \langle [ag \; \mathtt {pwr}] \rangle \langle [ag \; \mathtt {pwr}] \rangle \varphi$ for some $\varphi$. The truth condition for powers now says that $\exists P \in \mathcal {P}(m,ag) \text{ such } \text{ that } \forall h' \in P, \langle l, m, h' \rangle \models \langle [ag \; \mathtt {pwr}] \rangle \varphi$. Because of condition 2.b of Definition 4.2 we know that $P \not = \emptyset$. Then, because of moment determinacy of power, if $\langle l, m, h' \rangle \models \langle [ag \; \mathtt {pwr}] \rangle \varphi$ holds for some $h' \in P$ running through *m*, it holds for any *h* running through *m*. So, we immediately also have that $\langle l, m, h \rangle \models \langle [ag \; \mathtt {pwr}] \rangle \varphi$, which we needed to prove.

$$\begin{aligned} \begin{array}{rr} {(reduction\;of\;double\;power\;negation) } &{} \lnot \langle [ag \; \mathtt {pwr}] \rangle \lnot \langle [ag \; \mathtt {pwr}] \rangle \varphi \rightarrow \langle [ag \; \mathtt {pwr}] \rangle \varphi \\ \end{array} \end{aligned}$$

We will prove the contraposition $\lnot \langle [ag \; \mathtt {pwr}] \rangle \varphi \rightarrow \langle [ag \; \mathtt {pwr}] \rangle \lnot \langle [ag \; \mathtt {pwr}] \rangle \varphi$. Consider a situation $\langle l, m, h \rangle$ relative to a model $\langle L, M, H, \mathcal {L}, \mathcal {X}, \mathcal {H}, \mathcal {P}, \mathcal {O}, V \rangle$ such that $\langle l, m, h \rangle \models \lnot \langle [ag \; \mathtt {pwr}] \rangle \varphi$. This says that there is no *P* in $\mathcal {P}(m,ag)$ such that for all $h' \in P$ we have that $\langle l, m, h' \rangle \models \varphi$. (*) Because of moment determinacy of powers we have for any $h''$ through *m* that $\langle l, m, h'' \rangle \models \lnot \langle [ag \; \mathtt {pwr}] \rangle \varphi$. Now take an arbitrary $P'$ in $\mathcal {P}(m,ag)$. Such a $P'$ exists because of condition 2.b of Definition 4.2. From (*) it follows that also for all $h''$ in $P'$ we have that $\langle l, m, h'' \rangle \models \lnot \langle [ag \; \mathtt {pwr}] \rangle \varphi$. So, $P'$ is a witness for the fact that $\langle l, m, h \rangle \models \langle [ag \; \mathtt {pwr}] \rangle \lnot \langle [ag \; \mathtt {pwr}] \rangle \varphi$.

$$\begin{aligned} \begin{array}{rr} {(powers\;induce\;branching) } &{} \langle [ag \; \mathtt {pwr}] \rangle [\mathtt {X}] \varphi \rightarrow \langle \mathtt {H} \rangle [\mathtt {X}] [\mathtt {H}] \varphi \\ \end{array} \end{aligned}$$

Consider a situation $\langle l, m, h \rangle$ relative to a model $\langle L, M, H, \mathcal {L}, \mathcal {X}, \mathcal {H}, \mathcal {P}, \mathcal {O}, V \rangle$ such that $\langle l, m, h \rangle \models \langle [ag \; \mathtt {pwr}] \rangle [\mathtt {X}] \varphi$. It follows that $\exists P \in \mathcal {P}(m,ag)$ such that $(*) \forall h' \in P, \langle l, m, h' \rangle \models [\mathtt {X}] \varphi$. Take some $h'' \in P$. From condition 2.d of Definition 4.2 it follows that for all $h'''$ with $m \in h'''$ and $next_{h''}(m) = next_{h'''}(m)$, we have that $h''' \in P$. We know from (*) that for all such $h'''$, we have that $\langle l, next_{h'''}(m), h''' \rangle \models \varphi$. It follows that $\langle l, m, h'' \rangle \models [\mathtt {X}] [\mathtt {H}] \varphi$. But then we have that $\langle l, m, h \rangle \models \langle \mathtt {H} \rangle [\mathtt {X}] [\mathtt {H}] \varphi$.

*We skip the proofs for the opportunity schemas; they very closely follow the structure of the corresponding proofs for powers. *

$$\begin{aligned} \begin{array}{rr} {(opportunity-power\;reduction) } &{} \langle [\mathtt {oppt}] \rangle \langle [ag \; \mathtt {pwr}] \rangle \varphi \leftrightarrow \langle [ag \; \mathtt {pwr}] \rangle \varphi \\ \end{array} \end{aligned}$$

Consider a situation $\langle l, m, h \rangle$ relative to a model $\langle L, M, H, \mathcal {L}, \mathcal {X}, \mathcal {H}, \mathcal {P}, \mathcal {O}, V \rangle$ such that $\langle l, m, h \rangle \models \langle [\mathtt {oppt}] \rangle \langle [ag \; \mathtt {pwr}] \rangle \varphi$ (the left hand side of the schema is true). It follows that $\exists O \in \mathcal {O}(m,l)$ such that for all $h' \in O$ we have that $\langle l, m, h' \rangle \models \langle [ag \; \mathtt {pwr}] \rangle \varphi$. From condition 3.c of Definition 4.2 we know that $O \not = \emptyset$. So, let us say that $h'' \in O$. It follows that $\langle l, m, h'' \rangle \models \langle [ag \; \mathtt {pwr}] \rangle \varphi$. Now from moment determinacy of power we have that for any $h'''$ running through *m* it holds that $\langle l, m, h''' \rangle \models \langle [ag \; \mathtt {pwr}] \rangle \varphi$. So also $\langle l, m, h \rangle \models \langle [ag \; \mathtt {pwr}] \rangle \varphi$.

Consider a situation $\langle l, m, h \rangle$ relative to a model $\langle L, M, H, \mathcal {L}, \mathcal {X}, \mathcal {H}, \mathcal {P}, \mathcal {O}, V \rangle$ such that $\langle l, m, h \rangle \models \langle [ag \; \mathtt {pwr}] \rangle \varphi$ (the right hand side of the schema is true). Now from moment determinacy of power we have that for any $h''$ running through *m* it holds that $\langle l, m, h'' \rangle \models \langle [ag \; \mathtt {pwr}] \rangle \varphi$. From conditions 3.b and 3.c of Definition 4.2 it follows that $\exists O \in \mathcal {O}(m,l)$ such that $O \not = \emptyset$. From condition 3.a it follows that for any $h \in O$ we have that $m \in h$. But then *O* is witness to $\langle l, m, h \rangle \models \langle [\mathtt {oppt}] \rangle \langle [ag \; \mathtt {pwr}] \rangle \varphi$.

$$\begin{aligned} \begin{array}{ll} {{(power{\hbox {-}}opportunity~reduction)}} &{} \langle [ag \; \mathtt {pwr}] \rangle \langle [\mathtt {oppt}] \rangle \varphi \leftrightarrow \langle [\mathtt {oppt}] \rangle \varphi \\ \end{array} \end{aligned}$$

The proof structure is the same as for the previous property with the roles of powers and opportunities interchanged.

### Proofs for proposition 5.1

$$\begin{aligned} \begin{array}{ll} {\text {(agglomeration and monotonicity)}} &{} [ag \; \mathtt {Estit}] \varphi \wedge [ag \; \mathtt {Estit}] \psi \leftrightarrow [ag \; \mathtt {Estit}] (\varphi \wedge \psi ) \\ \end{array} \end{aligned}$$

Definition 5.2 item 2 states that situational h-effectivity functions interpreting modalities $[ag \; \mathtt {Estit}] \varphi$ map each situation to a (possibly empty) set of situations. $[ag \; \mathtt {Estit}] \varphi$ holds in a situation if and only if $\varphi$ holds in the situations mapped to. So, our situational h-effectivity functions are nothing more than classical Kripke frames. This means that as far as effectivity is concerned we deal with normal modal logic frames obeying both agglomeration and monotonicity (together equivalent to the Kripke axiom K). Proofs are trivial and to be found in any textbook on modal logic. $\square$

$$\begin{aligned} \begin{array}{ll} {(transitivity\;and\;Euclidicity) } &{} \text{ axioms } \text{4 } \text{ and } \text{5 } \text{ for } \text{ each } [ag \; \mathtt {Estit}] \\ \end{array} \end{aligned}$$

$[ag \; \mathtt {Estit}] \varphi \rightarrow [ag \; \mathtt {Estit}][ag \; \mathtt {Estit}] \varphi$ and $\lnot [ag \; \mathtt {Estit}] \varphi \rightarrow [ag \; \mathtt {Estit}] \lnot [ag \; \mathtt {Estit}] \varphi$ Follow directly from the fact that h-effectivity function outcomes are *sets*. If the set is empty (indicating that we are on a history interpreting a power or opportunity, but not a real posibilty), the axioms hold trivially.

$$\begin{aligned} \begin{array}{ll} {(conditional\;success) } &{} \langle ag \; \mathtt {Estit} \rangle \top \rightarrow ([ag \; \mathtt {Estit}] \varphi \rightarrow \varphi ) \\ \end{array} \end{aligned}$$

First consider situations where $\langle l, m, h \rangle \not \models \langle ag \; \mathtt {Estit} \rangle \top$. In that case we trivially have that $\langle l, m, h \rangle \models \langle ag \; \mathtt {Estit} \rangle \top \rightarrow ([ag \; \mathtt {Estit}] \varphi \rightarrow \varphi )$. Second consider situations where $\langle l, m, h \rangle \models \langle ag \; \mathtt {Estit} \rangle \top$. It follows that $\mathcal {E}(l,m,h,ag) \not = \emptyset$. Now from condition 2.b of Definition 5.2 it follows that in that case $h \in \mathcal {E}(l, m, h, ag)$. So, from $\langle l, m, h \rangle \models [ ag \; \mathtt {Estit} ] \varphi$ it follows that $\langle l, m, h \rangle \models \varphi$.

$$\begin{aligned} \begin{array}{ll} {(effectivity\;for\;historical\;necessities) } &{} [\mathtt {H}] \varphi \rightarrow [ag \; \mathtt {Estit}] \varphi \\ \end{array} \end{aligned}$$

$\langle l, m, h \rangle \models [\mathtt {H}] \varphi$ says that all situations $\langle l, m, h' \rangle$ based on histories running through *m* obey $\varphi$. Condition 2.c of Definition 5.2 says that h-effectivity functions interpreting modalities $[ag \; \mathtt {Estit}] \varphi$ in a situation $\langle l, m, h \rangle$ select subsets of the histories running through *m*; the modality evaluates to true if $\varphi$ holds relative to all histories in the set. So, it follows that $\langle l, m, h \rangle \models [ag \; \mathtt {Estit}] \varphi$.

$$\begin{aligned} \begin{array}{ll} {(effectivity\;requires\;power\;and\;opportunity) } &{} \langle \mathtt {H} \rangle [ag \; \mathtt {Estit}] \varphi \rightarrow \langle [ag \; \mathtt {pwr}] \rangle \varphi \wedge \langle [\mathtt {oppt}] \rangle \varphi \\ \end{array} \end{aligned}$$

Follows directly from conditions 2.d and 2.e in Definition 5.2 saying that if $\mathcal {E}(l, m, h, ag) \not = \emptyset$ then $\mathcal {E}(l, m, h, ag) \in \mathcal {P}(m,ag)$, and if $\mathcal {E}(l, m, h, ag) \not = \emptyset$ then $\mathcal {E}(l, m, h, ag) \in \mathcal {O}(l,m)$.

$$\begin{aligned} \begin{array}{ll} {(no\;choice\;between\;undivided\;histories) } &{} \langle ag \; \mathtt {Estit} \rangle \top \rightarrow ([ag \; \mathtt {Estit}] [\mathtt {X}] \varphi \rightarrow [\mathtt {X}] [\mathtt {H}] \varphi ) \\ \end{array} \end{aligned}$$

First consider situations where $\langle l, m, h \rangle \not \models \langle ag \; \mathtt {Estit} \rangle \top$. In that case we trivially have that $\langle l, m, h \rangle \models \langle ag \; \mathtt {Estit} \rangle \top \rightarrow ([ag \; \mathtt {Estit}] [\mathtt {X}] \varphi \rightarrow [\mathtt {X}] [\mathtt {H}] \varphi )$. Second consider situations where $\langle l, m, h \rangle \models \langle ag \; \mathtt {Estit} \rangle \top$. It follows that $\mathcal {E}(l,m,h,ag) \not = \emptyset$. from conditions 2.d and 2.e in Definition 5.2 it follows that $\mathcal {E}(l, m, h, ag) \in \mathcal {P}(m,ag) \cap \mathcal {O}(l,m)$. But, for this proof we only need either $\mathcal {E}(l, m, h, ag) \in \mathcal {P}(m,ag)$ or $\mathcal {E}(l, m, h, ag) \in \mathcal {O}(l,m)$ (we take the first). Since $\mathcal {E}(l, m, h, ag) \in \mathcal {P}(m,ag)$, from condition 2.d in Definition 4.2 we have that (*) for any $h' \in \mathcal {E}(l,m,h,ag)$ and $h'' \in H$ and $m \in h''$ and $next_{h'}(m) = next_{h''}(m)$ then $h'' \in \mathcal {E}(l,m,h,ag)$. We need to prove now that $\langle l, m, h \rangle \models [ag \; \mathtt {Estit}] [\mathtt {X}] \varphi \rightarrow [\mathtt {X}] [\mathtt {H}] \varphi$, and to that end we assume $\langle l, m, h \rangle \models [ag \; \mathtt {Estit}] [\mathtt {X}] \varphi$. It follows that for any $h' \in \mathcal {E}(l,m,h,ag)$ we have that $\langle l, m, h' \rangle \models [\mathtt {X}] \varphi$ and thus $\langle l, next_{h'}(m), h' \rangle \models \varphi$. Now consider any $h''$ with $m \in h''$ and $next_{h''}(m) = next_{h}(m)$. From (*) it follows that since $h \in \mathcal {E}(l,m,h,ag)$ (the succes condition in condition 2.b of Definition 5.2) we have that $h'' \in \mathcal {E}(l,m,h,ag)$ and thus $\langle l, next_{h''}(m), h'' \rangle \models \varphi$. Since this holds for any $h''$ we get that $\langle l, m, h \rangle \models [\mathtt {X}] [\mathtt {H}] \varphi$.

$$\begin{aligned} \begin{array}{ll} {(local\;real\;possibility) } &{} \langle \mathtt {H} \rangle \langle ag \; \mathtt {Estit} \rangle \top \\ \end{array} \end{aligned}$$

Condition f. of Definition 5.2 says that relative to any situation there is an $h \in H$ such that $\mathcal {E}(l, m, h, ag) \not = \emptyset$, which corresponds directly with the above schema.

$$\begin{aligned} \begin{array}{ll} {(shared\;space\;dependence) } &{} \bigwedge \limits _{ag_i \in Ags} \langle \mathtt {H} \rangle \langle \mathtt {L} \rangle (\langle ag \; \mathtt {Estit} \rangle \top \wedge [ag_i \; \mathtt {Estit}] \varphi _i) \rightarrow \\ &{} \langle \mathtt {H} \rangle \bigwedge \limits _{ag_i \in Ags} \langle \mathtt {L} \rangle (\langle ag \; \mathtt {Estit} \rangle \top \wedge [ag_i \; \mathtt {Estit}] \varphi _i) \\ \end{array} \end{aligned}$$

Take a situation $\langle l, m, h \rangle$ where $\langle l, m, h \rangle \models \bigwedge \nolimits _{ag_i \in Ags} \langle \mathtt {H} \rangle \langle \mathtt {L} \rangle (\langle ag \; \mathtt {Estit} \rangle \top \wedge [ag_i \; \mathtt {Estit}] \varphi _i)$. This says that for each $ag_i \in Ags$ there is a situation $\langle l_i, m, h_i \rangle$ such that $\mathcal {E}(l_i, m, h_i, ag_i) \not = \emptyset$ and for all $h' \in \mathcal {E}(l_i, m, h_i, ag_i)$ we have that $\langle l_i, m, h' \rangle \models \varphi _i$. Now, from condition 2.g of Definition 5.2 it follows that $\bigcap \nolimits _{0 < i \le \left| {Ags} \right| } \mathcal {E}(l_i, m, h_i, ag_i) \not = \emptyset$. But that means there is at least one $h'' \in \bigcap \nolimits _{0 < i \le \left| {Ags} \right| } \mathcal {E}(l_i, m, h_i, ag_i)$ and that for all $h''' \in \bigcap \nolimits _{0 < i \le \left| {Ags} \right| } \mathcal {E}(l_i, m, h_i, ag_i)$ it holds that $\langle l_i, m, h''' \rangle \models \varphi _i$. But then $\langle l, m, h \rangle \models \langle \mathtt {H} \rangle \bigwedge \nolimits _{ag_i \in Ags} \langle \mathtt {L} \rangle (\langle ag \; \mathtt {Estit} \rangle \top \wedge [ag_i \; \mathtt {Estit}] \varphi _i)$, with $h'''$ as a witnes for the existential modal quantifier $\langle \mathtt {H} \rangle$, and the $l_i$ as witnesses for each occurrence of the existential modal quantifier $\langle \mathtt {L} \rangle$.

## Footnotes

1 This example is from the third author of Linder et al. (1998).

2 There is the Dom tower as a meagre alternative, though.

3 To be even more precise: we consider agency at an abstraction level where the distinctions between motivational, informational and causal aspects do not matter; and this is also exactly the level Sergot aims at with his ‘unwitting’ agency.

4 We will here only consider Chellas’ version of such operators: $[ag \; \mathtt {Cstit}] \varphi$, where choice alternatives are not part of the truth condition (which is not a limitation for the logic, since we can define the availability of choice alternatives in the object language: $[ag \; \mathtt {dstit}] \varphi \equiv _{def} [ag \; \mathtt {Cstit}] \varphi \wedge \Diamond \lnot \varphi$).

5 We do believe in the relevance of quantum mechanics for understanding the phenomenon of agency, and for the theory of entanglement of distant particles quantum mechanics actually *conflicts* with relativity.

6 We think ‘branching time’ is a misnomer, as it is not time itself, but the space of possibilities through which it flows that branches, but we prefer to stick to established terminology.

7 In the sense explained by Tarski (1986).

8 This is certainly not the only possible view. An alternative is, for instance, to give time a less ‘derived’ identity, such that it can pass or branch independent of the properties true at moments. Or we can adopt a more idealistic view where time only passes and/or branches in our observations.

9 The semantic equivalence notion induced by this criterion is known in modal logic as ‘bisimulation’.

10 We do not believe that agents are the only entities that have the power to interfere with the branching of time. We just see no reason to deny the privilege to other natural phenomena (maybe animals, plants).

11 And when looking back we have a hard time believing that the branch we are on now was not special already in the past; maybe somewhat similar to how in early days it used to be that people had a hard time believing that our planet is not the centre of the universe.

12 We can imagine spaces that are completely decoupled from ours: parallel universes.

13 Note that such an elegant definition of space is not available to those who do not believe in the branching of time.

14 Note that we do not write ‘when’, to leave room for relativistic physics.

15 To be precise: this depends on a positive or negative velocity of the frame of reference in the direction of the line between Earth and Alpha Centauri.

16 The semantics would have to be reconsidered thoroughly. In a relativistic picture, it is not possible to evaluate against what we have called ‘situations’ because we do not have an absolute space frame to evaluate truth against. Therefore, like Belnap, we would have to evaluate against ‘point events’. It is unclear then if in such a picture a distinction between logical effects and physical effects makes sense.

17 In the meta-language we use the same symbols both as constant names and as variable names, and we assume universal quantification of unbound meta-variables.

18 Which again shows that ‘branching *time*’ is a misnomer, see footnote 6.

19 All meta variables not explicitly quantified are assumed to be universally quantified.

20 However, if they would, they would probably still be finitely axiomatizable, because the product logic $S5 \times S5 \times Alt.D$ is very likely to be ‘product matching’ (Gabbay and Shehtman 1998) which means it is axiomatisable using standard commutativity and confluency axioms (thanks to Agi Kurucz for pointing this out to me).

21 One example of this is Pólya’s theorem saying that a random walk in two dimensions is guaranteed to return to its origin, while a random walk in three dimensions has a chance of approximately only 0.34 to do so.

22 One can ask the reverse question: what *is* captured by three commutativity axioms for three pairs of dimensions in a three dimensional S5 set-up? The answer to this is ‘a kind of loosely coupled three dimensional space that is *not* a coordinate system’.

23 It is tempting to extend this picture to natural language and certain philosophical debates the actual impact of which seems hard to check.

24 It is an interesting fact that the behaviour in virtual worlds of computer games is mostly modelled along the lines of the theory of affordances and potentialities: objects in virtual environments typically have their own local animated behaviours attached to them (often explosions, collapses), and these are visualised as soon as they are directly or indirectly triggered by the player.

25 In the meta-language we use the same symbols both as constant names and as variable names, and we assume universal quantification of unbound meta-variables.

26 This is a standard modelling view from game theory where such functions are generally called ‘effectivity functions’. We avoid calling these functions ‘effectivity functions’ here, because in Sect. 5 we will define a notion of effectivity with a more precise meaning than in game theory. The technical notion ‘effectivity function’ can be said to hide a lot of conceptual structure that is worth bringing to the fore, like we aim to do in this article.

27 A real possibility is still only a possibility; there is not one possibility singled out as being ‘the’ most real or factual possibility. We remain strongly committed to the non-deterministic world-view.

28 It hardly can get more logical than that.

29 The ‘could have prevented’ condition is easily definable in *stit* formalisms.

30 All free meta-variables in these first-order conditions are assumed to be universally quantified; we do not make these quantifications explicit to keep the conditions readable.

31 Note we would have to get rid of the property $\langle [ag \; \mathtt {pwr}] \rangle \top$.

32 Maybe the difference between dependence and independence can be made more clear by sketching the analogy with multi-agent epistemic logic (van Ditmarsch et al. 2015). Making this comparison is more than observing a similarity in the mathematical structures; the *stit* concept of ‘indetermination’ and the epistemic concept of ‘uncertainty’ have much in common (and most compatibilists would likely say there is no difference at all). This is reflected by their modal semantics. In the case of epistemic logic, the possible worlds are the ones that are epistemically possible modulo the uncertainty admitted by the beliefs of an agent. In *stit* logic, the possible histories (that in this context play the role of possible worlds) are the ones that are possible modulo the indeterminacy admitted by the choice of an agent. Now, if in epistemic logic we would demand that intersections of sets of possible worlds of different agents are never empty (like we do for the choices in *stit*), then we would get *dependence* of beliefs: it would never be possible that if one agent beliefs *p*, another agent would belief $\lnot p$.

33 Note that essentially the difference between the two views is that in the traditional view the ‘choice cell’ (Horty’s terminology) itself is what the choice is, while in our view the properties necessitated by that choice cell are what the choice is. So, even in the well-studied context of *stit*-logic, we still have interpretation problems related to different views on act individuation.

34 A natural questions is then how to think about histories that start out ‘real’, then become ‘unreal’ (counter-factual), and then end up ‘real’ again. This is not something to worry about. Actually, it is the standard situation. Histories that stay ‘unreal’ continuously can only be like that if their counter-factuality is reinstated at every branching point they encounter, which corresponds to a nesting of counter-factuals seldom needed in reasoning contexts.

35 All meta variables not explicitly quantified are assumed to be universally quantified, that is, I will leave out universal quantifiers whenever their presence is obvious from the context.

36 One could argue that a logic of physical possibilities should not treat them as dependent entities, since only one possibility can be realised at the time. That is a valid, and natural view in so far non-agentive possibilities are concerned. But, note that here we talk about how what one agent can do has a bearing on what other agents can do at the same time.

37 Or, if we want to give the bomb some time to do its devastating work: use $[\mathtt {X}] p$ for painting the house and $[\mathtt {X}][\mathtt {L}] \lnot p$ for the result of the explosion.
